# Spatial immunoprofiling of the intratumoral and peritumoral tissue of renal cell carcinoma patients

**DOI:** 10.1038/s41379-021-00864-0

**Published:** 2021-07-02

**Authors:** Oscar Brück, Moon Hee Lee, Riku Turkki, Ilona Uski, Patrick Penttilä, Lassi Paavolainen, Panu Kovanen, Petrus Järvinen, Petri Bono, Teijo Pellinen, Satu Mustjoki, Anna Kreutzman

**Affiliations:** 1 Translational Immunology Research Program, University of Helsinki, Helsinki, Finland; 2 iCAN Digital Precision Cancer Medicine Flagship, Helsinki, Finland; 3 Hematology Research Unit Helsinki, University of Helsinki and Comprehensive Cancer Center, Helsinki University Hospital, Helsinki, Finland; 4 Comprehensive Cancer Center, Department of Hematology, Helsinki University Hospital, Helsinki, Finland; 5 Institute for Molecular Medicine Finland, HiLIFE, University of Helsinki, Helsinki, Finland; 6 Abdominal Center, Urology, Helsinki University Hospital and University of Helsinki, Helsinki, Finland; 7 Department of Pathology, HUSLAB, Helsinki University Hospital and University of Helsinki, Helsinki, Finland; 8 Comprehensive Cancer Center, Helsinki University Hospital and University of Helsinki, Helsinki, Finland; 9 Department of Clinical Chemistry and Hematology, University of Helsinki, Helsinki, Finland

**Keywords:** Renal cell carcinoma, Imaging the immune system, Immunohistochemistry

## Abstract

While the abundance and phenotype of tumor-infiltrating lymphocytes are linked with clinical survival, their spatial coordination and its clinical significance remain unclear. Here, we investigated the immune profile of intratumoral and peritumoral tissue of clear cell renal cell carcinoma patients (*n* = 64). We trained a cell classifier to detect lymphocytes from hematoxylin and eosin stained tissue slides. Using unsupervised classification, patients were further classified into immune cold, hot and excluded topographies reflecting lymphocyte abundance and localization. The immune topography distribution was further validated with The Cancer Genome Atlas digital image dataset. We showed association between *PBRM1* mutation and immune cold topography, *STAG1* mutation and immune hot topography and *BAP1* mutation and immune excluded topography. With quantitative multiplex immunohistochemistry we analyzed the expression of 23 lymphocyte markers in intratumoral and peritumoral tissue regions. To study spatial interactions, we developed an algorithm quantifying the proportion of adjacent immune cell pairs and their immunophenotypes. Immune excluded tumors were associated with superior overall survival (HR 0.19, *p* = 0.02) and less extensive metastasis. Intratumoral T cells were characterized with pronounced expression of immunological activation and exhaustion markers such as granzyme B, PD1, and LAG3. Immune cell interaction occurred most frequently in the intratumoral region and correlated with CD45RO expression. Moreover, high proportion of peritumoral CD45RO+ T cells predicted poor overall survival. In summary, intratumoral and peritumoral tissue regions represent distinct immunospatial profiles and are associated with clinicopathologic characteristics.

## Introduction

Clear cell renal cell carcinoma (ccRCC) constitutes the most common form of kidney cancer [1]. In ccRCC, Von Hippel-Lindau gene mutation and dysregulation lead to vascular endothelial growth factor (VEGF) and platelet-derived growth factor (PDGF) overproduction and constitutive oncogenic signaling [2, 3]. Moreover, genetic aberrations affecting the SWItch/Sucrose Non-Fermentable (*SWI/SNF*) chromatin remodeling complex such as the commonly mutated gene polybromo 1 (*PBRM1*) hamper multiple signaling pathways enhancing oncogenesis [3]. Nephrectomy represents the primary treatment modality for localized disease. Metastasis is detected in 20% of patients at diagnosis and manifests in another 20–30% at later timepoints [4]. Advanced disease is managed with systemic therapies, such as immune checkpoint inhibitors, tyrosine kinase inhibitors (TKIs) as well as mechanistic target of rapamycin complex 1 (mTORC1) inhibitors, and occasionally with metastasectomy [1, 4].

Among solid tumors, the highest lymphocyte infiltration quantified as transcriptomic cytolytic activity has been described in kidney cancers, notably ccRCC [5]. Upon activation, T cells produce CD25, sensitizing them to the mitogenic IL-2 cytokine and increase the cytolytic granzyme B (GrB) as well as immune checkpoint receptors programmed cell death protein (PD1), tumor necrosis factor receptor superfamily member 4 (OX40) and lymphocyte-activation gene 3 (LAG3) [6]. PD1 counter-regulates responses of activated lymphocytes, while LAG3 competes with the costimulation of helper T cells by binding class II human leukocyte antigen (HLA) [7, 8]. Persisting adaptive immune responses eventually become suppressed in processes known as immune senescence and exhaustion, which are translated into CD57 and T cell immunoglobulin and mucin-domain containing-3 (TIM3) expression, respectively [9, 10].

Immune topographies consist of spatial immune infiltration patterns defined by histological examination [11, 12]. The immune hot topography represents an inflamed phenotype characterized by high intratumoral immune infiltration contrary to immune cold tumors, which are devoid of immune cells. In turn, immune excluded tumors are defined by high peritumoral but low intratumoral immune cell accumulation. Previous studies have shown that the baseline immune profile such as the immune topography predicts immunotherapy response [13–16]. More precisely, immune cold ccRCC with mutated *PBRM1* and immune hot tumors with del(9p21.3) have retrospectively been shown to predict superior responses to anti-PD1 blockade [17]. Yet, the significance of immune topographies and separate intratumoral and peritumoral tumor regions in RCC remain unclear.

In this study, we aimed to characterize and compare the spatial profile of the intratumoral and peritumoral tissues. We classified RCC patients into immune topographies and combined these classes with phenotype-based immune profiles defined with quantitative multiplex immunohistochemistry (mIHC). We computed intercellular distances to combine immunologic spatiality with immunophenotypes, immune topographies and clinical characteristics. Finally, we studied how immune cell abundance, location and interaction-dependent immunophenotypes are associated with prognosis.

## Materials and methods

### Patient selection

A total of 136 clear cell RCC patients underwent nephrectomy at diagnosis and received TKI sunitinib as first-line systemic therapy for advanced disease either at diagnosis or after disease progression at the Helsinki University Hospital (HUH) Comprehensive Cancer Center between October 18, 2006 and December 31, 2014 [18]. Fresh nephrectomy samples were formalin-fixed and paraffin-embedded along routine clinical diagnostics in the Department of Pathology, HUSLAB. From these, we included only patients (*n* = 64) with a nephrectomy sample extending from the intratumoral tissue over the peritumoral margin (Supplementary Fig. 1A and Table 1). As control, we analyzed normal renal tissue extracted macroscopically apart from IT and PT regions of 11 nephrectomy samples. The study complied with the Declaration of Helsinki and the HUH ethics committee.

**Table 1:** Patient characteristics.

Clinical variables	Patients (*n* = 64)
Age (years), median [range]	64.9 [41.2–83.2]
Gender (male)	37 (58%)
T	
1	15 (23%)
2	10 (16%)
3	37 (58%)
4	2 (3%)
N	
0	47 (73%)
1	17 (27%)
M	
0	34 (53%)
1	30 (47%)
Stage	
1	8 (13%)
2	9 (14%)
3	16 (25%)
4	31 (48%)
Fuhrman grade	
1	4 (6%)
2	24 (39%)
3	29 (45%)
4	6 (9%)
Mortality in 5 years	36 (56%)
Sunitinib response (RECIST criteria)	
Partial response	17 (27%)
Stable disease	35 (55%)
Progressive disease	6 (9%)
Not defined	6 (9%)
Sunitinib treatment time (days), median [range]	255 [13–2028]
Sunitinib dosage	
25 mg	17 (27%)
37 mg	31 (48%)
50 mg	16 (25%)
Sunitinib dose reduction	31 (48%)
Sunitinib dose escalation	22 (34%)
Progression-free survival events	
Treatment failure	32 (43%)
Censored, end of follow-up	13 (18%)
Censored, side effects	18 (24%)
Censored, request by the patient	1 (1%)
Treatment prior to sunitinib	
Targeted therapy	0 (0%)
Interferon-α	9 (14%)
MSKCC risk class	
Low	14 (22%)
Intermediate	43 (67%)
High	7 (11%)

Overall survival was defined as the time from diagnosis to death by censoring patients alive at their last follow-up date. Sunitinib treatment response was evaluated according to Response Evaluation Criteria in Solid Tumors 1.0 (RECIST) with computed tomography at 8–12-week intervals after treatment start until censoring or end of treatment.

### Lymphocyte detection

Nephrectomy tissue slides stained for H&E were digitized at 0.22 µm/pixel resolution with the Zeiss Plan-Apochromat 20x objective and Pannoramic P250 Flash II histological scanner (3DHistech). Intratumoral (IT) and peritumoral (PT) regions were manually annotated. The PT region was defined as the stromal border interiorly limited by the cancerous IT parenchymal tissue and exteriorly by normal renal glomerular and tubular tissue. A lymphocyte-detecting classifier was trained with the random trees algorithm using examples from 10 H&E-stained nephrectomy images and applied to all detected cells in both IT and PT regions. The lymphocyte cell proportion correlated with the lymphocyte proportion by area (Supplementary Fig. 1B). Tissue annotation, cell detection and lymphocyte classification were performed in the graphical image analysis environment QuPath [19].

### Color normalization

To validate the proportions of immune topographies, we analyzed H&E stained digital images of The Cancer Genome Atlas (TCGA) ccRCC pathology dataset. As these originate from multiple clinical centers with different tissue processing, H&E staining and slide digitization protocols, the color distribution varied substantially between samples. Therefore, we employed a structure-preserving color normalization pipeline based on sparse non-negative matrix factorization that has been modified from the original method described by Vahadane et al. (Supplementary Fig. 2A, B) [20, 21]. Briefly, each pixel represents a mixture of hematoxylin and eosin. These are first separated and then adapted to the stain concentration of a reference image. After careful assessment, we selected TCGA sample TCGA-B0-4691-01Z-00-DX1 as reference (Supplementary Fig. 2C). As the normalization does not perform well to image with highly deviant color distributions, we manually verified results and included only successful normalized images (Supplementary Fig. 2A). Immune cell proportions and topographies for the Helsinki and TCGA ccRCC datasets are provided in Supplementary Table 1 and results in Supplementary Fig. 2D.

### Somatic mutation data

We collected somatic exome mutation for most commonly altered genes from the supplementary data deposited part of the landmark TCGA pan-renal study [22]. Owing to limitations in sample size, we focused only on genes mutated ≥5% of patients of the TCGA ccRCC dataset with immune topography data. The sequencing data was available for 97/113 of these patients. The data included single nucleotide variants and indels, which had been called based on the results of six algorithms (MuTect, MuSE, Pindel, Somatic Sniper, VarScan2 and Radia).

### Tissue microarrays (TMAs)

**Fig. 1:**
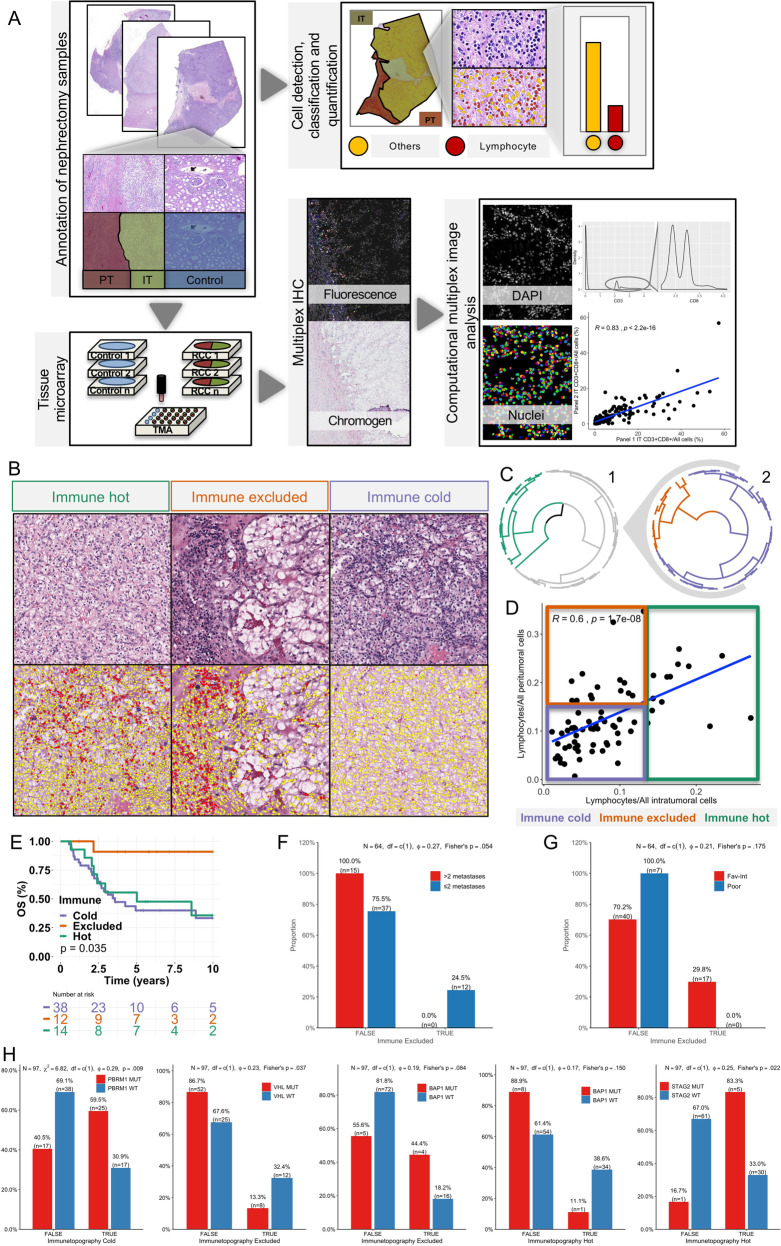
Defining immune topographies. **A** Overview of the study. Nephrectomy samples of renal cell carcinoma (RCC) patients were reconstructed into tissue microarrays (TMA) and stained with H&E and multiplex immunohistochemistry (mIHC). The intratumoral (IT), peritumoral (PT) and normal control tissue were examined separately. A cell classifier was developed to detect lymphocytes from H&E morphology. Cells were detected and classified in mIHC images based on marker intensity and co-localization. **B** Representative H&E images of different immune topographies (upper row) and corresponding cell detection and classification results (bottom row). Lymphocytes are red-colored and non-lymphocytes yellow-colored. **C** Two-phase clustering of (1) IT lymphocyte proportion and (2) the arithmetic difference of IT and PT lymphocyte proportion with Euclidean distance. **D** Linear regression of IT and PT lymphocyte proportions (plot) and Spearman correlation (left upper corner). **E** Kaplan–Meier visualization of overall survival from diagnosis by immune topographies. **F** Characterization of excluded immune topography by number of metastases and **G** MSKCC risk group with barplots and Fisher’s test. **H** Barplots of genetic alterations in most commonly mutated genes and tumors with an immune hot, immune excluded, and immune cold topography.

TMA blocks were mounted using 2 mm cores from the invasive margin of FFPE nephrectomy tissue blocks consisting of both PT and IT regions. Additional dual cores from 11 macroscopically and microscopically confirmed non-malignant regions of nephrectomy samples were selected to represent healthy kidney tissue (Fig. 1A).

### Multiplex immunohistochemistry (mIHC)

The mIHC method is founded on an iterative application of antibody-based stainings, resulting in 5-plex fluorescence and 3-plex chromogenic IHC (Fig. 1A). Primary antibody mIHC panels included a total of 23 different markers to detect cancer, T, NK and CD16 + myeloid cells and their immune checkpoint and activation phenotypes (Supplementary Tables 2–3). Technical details are described in previous publications [23, 24].

#### General

TMA blocks were cut in 4 µm sections on Superfrost objective slides (Kindler O Gmbh). After peroxide block, antibody application, and fluorochrome reaction, slides were washed three times with 0.1% Tween-20 diluted in 10 mM Tris-HCL buffered saline pH 7.4.

#### Tissue preparation

After tissue deparaffinization in xylene and rehydration in graded ethanol series, heat-induced epitope retrieval (HIER) was performed in 10 mM Tris-HCl–1 mM EDTA buffer (pH 9) in +99 °C for 20 min (PT Module, Thermo Fisher Scientific). Peroxide activity was blocked with 0.9% H_2_O_2_ for 15 min, and subsequently applied with 10% normal goat serum (TBS-NGS) for 15 min.

#### Antibody testing

Antibodies were evaluated based on their occurrence in previous studies and we prioritized monoclonal antibodies. For each marker, we assessed the staining patterns of one or multiple antibodies in lymphoid and hematopoietic tissues (lymph node and bone marrow), non-immunogenic tissue (brain tissue) and epithelial tissue (gut, healthy kidney tissue and renal cell carcinoma). We required that antibodies detecting immune markers are enriched in lymphoid and hematopoietic tissues and are colocalized in immune cells. We ensured that antibodies detected with fluorescent probes denatured in HIER to use them in multiplex setting. Moreover, for CD3, CD8, PD-L1, PD1, TIM3, LAG3, GrB and OX40 markers the proportion of antibody-stained cells have been correlated with their gene expression in previous studies in diffuse large B-cell lymphoma and testicular lymphoma tissues [25, 26]. We also confirmed that IHC staining with chromogen or fluorescent probes produced consistent results (Supplementary Fig. 3A).

#### Staining

Primary antibodies diluted in protein blocking solution according to Supplementary Table 3, and secondary anti-mouse or anti-rabbit horseradish peroxidase-conjugated (HRP; Immunologic) antibodies 1:1 in washing buffer were applied for 1 h 45 min and 45 min, respectively. Tyramide signal was amplified (TSA; PerkinElmer) for 10 min. Primary antibodies and HRP activity were inactivated with HIER. The peroxide and protein block steps were repeated. The primary antibody and its matching HRP-conjugated secondary antibody diluted 1:5 in washing buffer were added and TSA signal amplified. We repeated HIER, peroxide block and protein block as before and incubated the slides with two additional primary antibodies immunized in different species overnight in +4 °C. AlexaFluor647 and AlexaFluor750 fluorochrome-conjugated secondary antibodies (Thermo Fisher Scientific) diluted 1:150 in washing buffer (45 min) and 4′,6-diamidino-2-phenylindole counterstain (Dapi, Roche) diluted in 1:250 in TBS (15 min) were applied. Last, ProLong Gold (Thermo Fisher Scientific) was used to coverslip slides.

After fluorescence imaging, atraumatic coverslip detaching was ensured by incubating slides in washing buffer overnight in +4 °C. HIER, peroxide block and protein block were repeated as previously. We added primary antibodies immunized in separate species and species-matching alkaline phosphatase (AP) and HRP-conjugated secondary antibodies (Immunologic). Antibodies were detected with VinaGreen (Biocare Medical), then with Liquid Permanent Red (Dako) chromogens. To counterstain nuclei, Mayer’s hematoxylin diluted 1:10 in H_2_O was applied for 1 min. Slides were washed in H_2_O (30 s) after each staining reaction. Finally, slides were coverslipped with Pertex mounting medium.

### Imaging

Fluorescent and brightfield mIHC images were acquired with the AxioImager.Z2 (Zeiss) microscope supplemented with Zeiss Plan-Apochromat 20x objective, and CoolCube1 CCD camera (MetaSystems). Scanned images were converted to JPEG2000 format (95% quality).

### Image preprocessing

RGB colors were deconvolved from brightfield chromogen stainings [27]. Mean fluorescent and brightfield images were 8-fold downscaled and each spots registered using two-dimensional phase correlation method [28]. The staining quality was visually assessed, and a few unfocused images were eliminated from the analysis.

### Image analysis

Spots were manually separated into IT and PT regions guided by hematoxylin counterstain (Fig. 1A). Cell segmentation and intensity measurements were carried out using adaptive Otsu thresholding and upper quartile intensity of grayscaled dapi-stained images with the image analysis platform CellProfiler 2.1.2 (Fig. 1A) [29]. Clumped cells were separated based on staining intensity. Tissue cores with fewer than 1500 cells were eliminated from the analysis.

Cell classification was carried out by means of marker intensity and co-localization of multiple antibodies (Supplementary Table 4). Immune cell types were quantified as proportion to all cells (e.g., CD3+CD8+ cell count to total cell count) or as proportion to an immunophenotype defined by 1–2 markers to the cell type of interest (e.g., CD3+CD8+/PD1+LAG3+ corresponds to PD1+LAG3+ cell proportion of CD3+CD8+ cells). Immune spatial analysis was performed by computing the Euclidean distance between CD3+, CD3+CD4+, CD3+CD8+, CD2+CD3- and CD16+ cell centrum. Cells within a distance of 100 pixels equaling to 22 μm were defined as interacting cells [30]

$$d_{a_jb_k} = \sqrt {\left( {x_{a_j} - x_{b_k}} \right)^2 + \left( {y_{a_j} - y_{b_k}} \right)^2} {\it{,}}$$

where $d_{a_jb_k}$ represents the spatial distance of cell *a*_*j*_ and *b*_*k*_ located at ($x_{a_j},y_{a_j}$) and ($x_{b_k},y_{b_k}$) respectively. The cell interaction values for cells *a*_*j*_ and *b*_*k*_ were categorized as $i_{a_jb_k}$ into values 0 or 1 as follows

$$i_{a_jb_k} = \left\{ {\begin{array}{*{20}{c}} {1,d_{a_jb_k} \le 100} \\ {0,d_{a_jb_k} \,> \, 100} \end{array}} \right.{\it{,}}$$

In a sample with *m* number of cells *a* and *n* number of cells *b*, the interaction frequency for cells *a* and *b* is the sum of their categorized cellular interaction vectors

$$i_{ab} = \mathop {\sum }\limits_{j = 1}^m \mathop {\sum }\limits_{k = 1}^n i_{j,k} = i_{1,1} + i_{1,2} + \ldots + i_{m,n}{\it{,}}$$

where $i_{ab}$ represents the sum of all interactive cell pair *a* and *b* in one sample, and $j,k$ belong to index sets $j \in 1, \ldots ,m$and $k \in 1, \ldots ,n$. The sample-level interaction index $I_{ab}$ normalizes interaction frequencies by the proportion of the examined cell pair $a$ and $b$, defined as

$$I_{ab} = \frac{{i_{ab}}}{{\sqrt {\mathop {\sum }\nolimits^ {ab}} /\mathop {\sum }\nolimits^ c}}{\it{,}}$$

in a sample with total cell number $c$.

### Statistical analysis

We used the unpaired two-tailed Wilcoxon signed-rank test to compare continuous variables in two groups, and Kruskal–Wallis test in three groups. Benjamini–Hochberg’s method was used to correct for *p*-values [31]. Categorical variables were compared with the chi-square test (frequency for each variable >5) or Fisher’s exact test (frequency for any variable ≤5). Continuous variables were correlated with Spearman’s rank correlation coefficient. Heatmaps and immune topographies were clustered by Spearman correlation distance and Ward D2 linkage. For heatmaps, data were normalized with median-centering and max-scaling. Cox regression analysis (log-rank test) was used for survival analyses. All statistical analyses were performed with R v3.3.3.

## Results

### Clustering of immune cell quantities by their location defines immune topographies

By examining H&E stained tumor slides from RCC patients that underwent nephrectomy (*n* = 64), we observed recurrent IT and surrounding PT entities by their distinct tissue textures (Fig. 1A, Supplementary Fig. 1A, and Table 1). We developed a classifier detecting lymphocytes based on their conspicuous dark nuclei and little cytoplasm (Supplementary Fig. 3B). We observed a median PT lymphocyte proportion of 11.2% from all cells compared to 7.6% inside the IT regions (*p* < 0.001; Supplementary Fig. 3C). To determine immune topographies, we performed an unsupervised two-phase clustering on IT immune cell infiltration using Euclidean distance to first define patients with an immune hot topography (*n* = 14, Fig. 1B–D). Immune cold (*n* = 38) and excluded (*n* = 12) samples were distinguished by computing the Euclidean distance on the subtraction of PT and IT lymphocyte densities (Fig. 1C).

To validate the unsupervised immune topography classification platform, we examined H&E stained digital slides from TCGA diagnostic ccRCC pathology archive (Supplementary Fig. 2A). We selected only slides with distinct intratumoral and peritumoral tissue regions (*n* = 194). The cell detection and classification algorithm we employed did not perform well on slides with markedly deviant color distribution. Therefore, we normalized these with a structure-preserving color normalization method where deconvoluted colors are matched to a reference staining (Supplementary Fig. 2B, C). We performed then unsupervised immune topography clustering for the final 113 images for which normalization was successful. While the proportion of immune hot tumors was higher in the TCGA dataset (36.3% vs. 21.9%) and the proportion of immune cold tumors lower (43.4% vs. 59.4%), no significant differences were observed (Chi^2^ = 0.09, Supplementary Fig. 2D).

To further interrogate the pathogenomic background of immune topographies, we associated these with the somatic variants in genes altered in ≥5% of TCGA ccRCC patients. First, we demonstrated that genes *VHL* (62%) and *PBRM1* (43%) were the most commonly altered in line with previous reports (Supplementary Fig. 4A) [3, 32]. The total number of mutations was not associated with any particular immune topography (Supplementary Fig. 4B). When examining individual genes, we observed altered *PBRM1* to be associated with a cold immune topography (25/42 vs. 17/55, *p* = 0.009, *X*^2^ test; Fig. 1H and Supplementary Fig. 4C). The finding is consistent with previous reports indicating deficient *PBRM1* in ccRCC to induce a non-immunogenic tumor and resistance to immune checkpoint inhibitors [17, 32]. Patients with an immune excluded topography harbored less mutations in gene *VHL* (8/60 vs. 12/37, *p* = 0.04, Fisher’s test) and more in the *BAP1* gene (4/20 vs. 7/72, *p* = 0.08, Fisher’s test; Fig. 1H and Supplementary Fig. 4D). Instead, tumors with a hot immune topography lacked mutations in the *BAP1* gene (1/10 vs. 34/88, *p* = 0.15, Fisher’s test) but were enriched with mutations in the *STAG2* gene (5/6 vs. 30/91, *p* = 0.08, Fisher’s test; Fig. 1H and Supplementary Fig. 4E). In summary, the findings emphasize that rather than the total mutation burden, alterations in distinct genes are associated with the formation of immune topographies and could help to identify patients benefitting from immunotherapies.

Next, we studied prognostics related to immune cell infiltration and topography. Immune cell infiltration in the IT and PT regions did not stratify patients by survival (Supplementary Fig. 5A). However, patients with an immune excluded contexture were observed to share strikingly superior overall survival (HR 0.11, 0.015–0.82 95%CI *p* = 0.031, Cox regression) but not progression-free survival compared to immune hot and cold patients (Fig. 1E and Supplementary Fig. 5B). Patients with immune excluded tumors were observed to have fewer metastatic organs explaining their survival benefit and none of them were grouped into poor Memorial Sloan Kettering Cancer Center (MSKCC) risk score (Fig. 1F, G). Yet, no differences were noted in the histological grade, tumor stage, nor patient age, suggesting prognostic advantage independent from other previously known risk factors (Supplementary Fig. 5C–E). We also compared IT and PT tumor-infiltrating lymphocyte (TIL) fractions by histological grade, tumor stage, patient age, number of metastasis and MSKCC score, and observed low PT TIL proportion to be associated with low MSKCC score (Supplementary Fig. 5F, G).

### Quantitative immunohistochemistry reveals location-dependent lymphocyte immunoprofiles

**Fig. 2:**
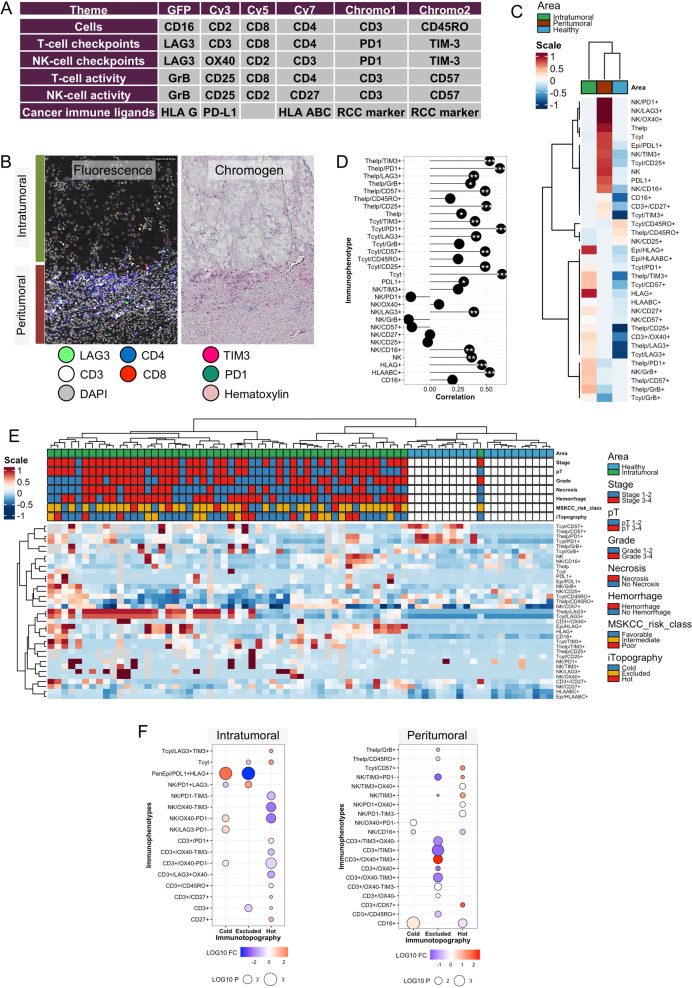
Characterization of immune contextures. **A** Panel design used in multiplex immunohistochemistry (mIHC). GFP, Cy3, Cy5, and Cy7 represents fluorescence channels and Chromo1 and Chromo2 chromogenic channels. **B** Representative fluorescence and chromogen staining between intratumoral (IT) and peritumoral (PT) tissues. **C** Contexture-level heatmap of immunophenotypes in IT, PT, and control tissues with median-averaged immune cell proportions. Clustering has been computed with the Euclidean distance of immunophenotype correlations (Spearman). **D** Spearman correlation between IT and PT immunophenotypes. Significance: ***p < 0.001, **p < 0.01, *p < 0.05. **E** Patient-level heatmap of immunophenotypes in IT and control tissues. Clustering has been computed with the Euclidean distance of immunophenotype correlations. **F** Balloonplot visualizing the log10 fold-change difference of immunophenotype proportions between immune topographies. Each topography has been compared to the pooled group of other topographies. The balloon size corresponds to the *p*-value (Wilcoxon test). Only immunophenotypes differing in any comparison (*p* < 0.05) are shown.

Next, we hypothesized that IT and PT regions could be immunologically distinct from each other and healthy renal tissue (*n* = 11). We designed panels consisting of 6 antibodies to quantify T, NK, and CD16+ myeloid cells and their immunophenotypes (Fig. 1A and Fig. 2A, B). By applying mIHC panels on consecutive TMA slides and analyzing separately IT, PT, and control tissue regions, we classified a total of 7.6 million cells with high interslide reproducibility (*R* = 0.83, *p* < 0.001 for CD3+CD8+ cytotoxic T cells in sequential slides).

When examining average-aggregated immune profiles, we noted an enrichment of T (helper and cytotoxic) and NK cells in the PT region (Fig. 2C). All immune cell populations and immunophenotypic markers were upregulated in both IT and PT areas compared to normal renal tissue, except for CD45RO, possibly representing tissue-resident memory T cells of the normal kidney tissue immune homeostasis (Fig. 2C) [33]. Multiple T-cell markers associated with immune activation or exhaustion, such as GrB, OX40, PD1, CD57, and LAG3 were concentrated in the IT region compared to the PT region [34]. Contrarily, the highest PD1, LAG3, and OX40 expression in NK cells and total PDL1 expression were observed in the PT region.

To quantify the similarity between immune contextures, we correlated IT and PT immune profiles at the patient-level (Fig. 2D). The median Spearman correlation coefficient of individual immune subtypes between IT and PT regions was 0.37. Higher correlations were observed for T-cell phenotypes (*R* = 0.45) and immune ligands (*R* = 0.46), contrary to NK cell markers (*R* = 0.04). As expected, correlation between proportions of IT and PT T-cell phenotype was driven by multiple markers (Supplementary Fig. 6A). However, only the fraction of OX40+ and CD16+ NK cells correlated between IT and PT regions.

Next, we examined individual patients in detail. LAG3 expression in T cells was observed to drive hierarchical clustering notably in the IT region, but did not share mutual expression patterns with PD1, TIM3 or OX40, possibly reflecting a difference in regulation (Fig. 2E and Supplementary Fig. 6B–D). Antigen-presenting class I HLA-ABC was upregulated both in PT and IT regions compared to control tissue (Fig. 2E). While HLA-G expression was unnoted in control and PT tissues, ~1/3 of tumors stained positive for HLA-G representing a potentially unique mechanism to escape immune surveillance (Fig. 2E). High HLA-G expression was enriched in patients of poor MSKCC risk class but did not associate with patient age, histological grade, metastasis status, TNM status, or immune topographies (Supplementary Fig. 7A–H). When examining immune cell populations, high HLA-G expression was affiliated with elevated IT NK, helper, and cytotoxic T-cell proportion but not with corresponding PT cell quantities (Supplementary Fig. 8A, B). Similar positive association between PD-L1 expression and high IT but not PT NK, helper, and cytotoxic T-cell proportion were noted (Supplementary Fig. 8C, D). In line, the expression of HLA-G and PD-L1 co-occurred potentially reflecting common regulatory pathways (Supplementary Fig. 8E) [35, 36].

### Immune topographies are characterized by distinct immune profiles

We hypothesized that immune topographies would differ by their IT and PT phenotypic composition. PD-L1+HLA-G+ cancer cells were enriched in IT immune cold samples, while depleted from IT immune excluded samples (Fig. 2F). Immune cold tumors were also observed to harbor PT CD16+ myeloid cells, which have been characterized as the principal cell-of-origin expressing PD-L1 in non-small-cell lung carcinoma and promoting immune evasion [37].

Immune hot and cold tumors differed in multiple immunophenotypes (Fig. 2F). IT cytotoxic T cells and CD45RO+ memory helper T cells were more numerous in immune hot tumors. Moreover, these tumors were characterized by T and NK cells expressing immune checkpoint receptors PD1, TIM3, OX40 and LAG3 as opposed to immune hot tumors. Both immune excluded and hot topographies were characterized with high PT immune infiltrate in the H&E staining analysis (Supplementary Fig. 9A). Yet, the PT T-cell immunophenotype of immune excluded tumors was marked with low expression of TIM3 and OX40 checkpoint receptors and CD45RO, potentially reflecting an unprimed immune signature (Fig. 2F).

### Interaction indexes reveal common spatially adjacent cell pairs

**Fig. 3:**
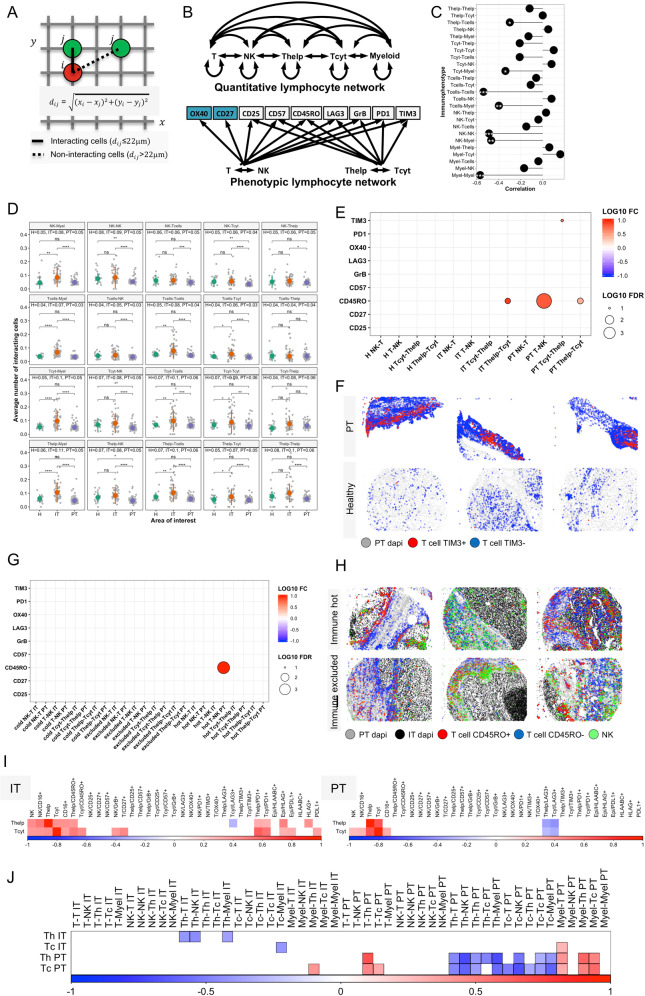
Spatial immune cell network. **A** Visualization of the Euclidean distance computed for each cell pair. **B** Visualization of the quantitative lymphocyte cellular (upper) and phenotypic (lower) network. Arrows in the cellular network represents individual computed comparisons, and in the phenotypic network the immunophenotypes compared between (non-)interacting immune cells. **C** Spearman correlation of interacting immune cell pairs between IT and PT regions. **D** Panel of comparisons of the cellular interaction frequency by intratumoral (IT), peritumoral (PT), and healthy (H) tissues. **E** Comparison of immunophenotypes by immune contexture (H, IT, PT) and interactive immune cell pair. Immunophenotypes are reported for the first immune cell by order. The fold-change (FC) has been calculated as the 10-fold logarithmic immunophenotype expression difference between interacting and non-interacting cells. For instance, “PT T-NK” represents immunophenotypes calculated for T cells interacting with NK cells in peritumoral renal tissue, and the red circle visualizes higher proportion of TIM3 expression in interacting vs. non-interacting T cells. **F** Digital staining for visualizing the location of TIM3+ T cells in PT and healthy renal tissue. IT tissues have been omitted for clarification. **G** Comparison of immunophenotypes by immune topography and interactive immune cell pair. The FC represents the 10-fold logarithmic immunophenotype expression difference between interacting and non-interacting cells. Immunophenotypes are reported for the first immune cell by order. **H** Digital staining for visualizing the location of CD45RO+ T cells and NK cells in immune hot and excluded renal tissues. **I** Spearman correlation matrix of intratumoral (IT) and peritumoral (PT) T-cell subsets. **J** Spearman correlation matrix between the proportion (rows) of IT and PT cytotoxic (Tc) and helper T cells (Th) and proportions of cell interactions (columns). The color scaling represents the correlation coefficient (red positive, blue negative). *p*-values have been adjusted with Benjamin & Hochberg correction. Only significant correlations (adjusted *p*-value < 0.05) are color-labeled. Significance: ***p < 0.001, **p < 0.01, *p < 0.05.

We hypothesized that studying the spatial hot spots of immune cells would indicate the location of occurring immune responses. Intercellular distances between T and myeloid cells <25 μm or the equivalent of 2–3 cell diameters have been associated with adaptive T-cell responses [30]. Therefore, interaction was defined as cell pairs within a radius of 100 pixels corresponding to 22 μm (Fig. 3A). We developed an algorithm calculating cell interaction indexes defined as the inclination of distinct cell pairs to occur in close spatial proximity. Indexes were normalized by the proportions of interacting cell pairs to avoid bias due to differences in immune cell proportions. Given the design of the cell panel, we calculated the interaction of all T, helper T, cytotoxic T, NK, and CD16+ myeloid cells (Fig. 3B).

Interaction indexes between IT and PT contextures demonstrated regionally distinct spatial conformation (Fig. 3C). Interaction indexes of T, NK, and CD16+ myeloid cells were observed to correlate negatively, signifying that their interactions in either the IT or PT region would imply a lack of interaction in the other region. Moreover, the IT region was associated with a higher interaction index than PT or healthy regions (Fig. 3D). CD16+ cells interacted more avidly with IT lymphocytes compared to PT and healthy areas. Furthermore, T and NK cells were observed to interact more commonly with each other in the IT than in the PT region.

The PT interaction index was lower between lymphocytes and CD16+ myeloid cells, as well as between cytotoxic T cells and other T cells than in the normal tissue (Fig. 3D). Given its high lymphocyte content, immunophenotypically non-exhausted profile, and lack of interaction, PT lymphocytes could be physically hindered from recognizing cancer cells and represent an immunologically inoperative reserve. By examining H&E tissue texture, we frequently observed prominent perpendicular orientation and deeper eosinophilic staining in the PT fibrous stroma, suggesting possibly denser, or qualitatively distinct collagen fibers (Supplementary Fig. 9B). We also compared the frequencies of interacting IT or PT immune cells in immune cold, hot and excluded tumors, but did not discern any differences (Supplementary Fig. 9C, D).

### Memory T cells frequently engage in cellular interaction

Next, we investigated the association of immunophenotype expression with cellular interaction. The design of panels 1–5 helped us to compare the immunophenotypes of interacting and non-interacting immune cells separately by their tissue region and tumor immune topography (Fig. 3B). We observed consistent positive correlation between IT and PT immunophenotype proportions when comparing immune cells by their interaction status, signifying that if an immunophenotype is positive in interacting IT cells, then a similar phenotype would also occur in interacting PT cells (Supplementary Fig. 10). Interacting PT T cells expressed higher levels of TIM3 than non-interacting T cells (Fig. 3E). T cells in control tissue adjacent to NK and helper T cells were associated with lower TIM3 expression. To demonstrate the physical proximity associated with PT TIM3 expression, we digitally reproduced the mIHC stainings by mapping cells according to their phenotype and location (Fig. 3F). The findings suggest that the tissue contexture imposes diverse effects on immune profiles depending on their interaction status.

Higher CD45RO expression was noted especially in IT and PT helper T cells interacting with cytotoxic T cells but not in control tissue (Fig. 3E). The isoform switch from CD45RA to CD45RO reflects the differentiation of primed T cells into avidly proliferating and cytolytic memory subtypes [6]. When comparing immune topographies, we observed that CD45RO expression was elevated in T cells interacting with NK cells notably in conjunction of immune hot tumors indicating a link between immune cell phenotype, interaction and immune topography (Fig. 3G, H).

### T-cell infiltration and checkpoint expression are characterized by contexture-dependent immunologic signatures

Next, we studied immune profiles associated with high T-cell infiltration and immune checkpoint expression as these have been suggested to predict response to immuno-oncological therapies [38]. In the IT region, T-cell infiltration was associated with more frequent NK, CD16+ myeloid cell and CD45RO+ and PD1+ T-cell proportions and higher expression of PD-L1 and HLA-G (Fig. 3I). Conversely, high PT T-cell enrichment correlated with high PT NK and CD16+ cell densities but low PT lymphocytic LAG3 expression. We also observed associative patterns between T-cell infiltration and spatial immune network. Abundant PT T cells correlated with low PT T-cell interaction (Fig. 3J). Elevated PT T cells were also linked with T and CD16+ myeloid cell proximity in the PT region. However, similar patterns between IT T-cell density and cell interaction were not noted.

Next, we examined immunologic profiles associated with PD1+TIM3+ and LAG3+TIM3+ T cells previously suggested to represent states of terminal exhaustion [39]. We observed multiple phenotypic associations to be distinct for PD1+TIM3+ and LAG3+TIM3+ subtypes (Supplementary Fig. 11A). IT and PT PD1+TIM3+ T cells were more frequent in tumors with CD45RO+ T cells and PDL1+ expression. Instead, LAG3+TIM3+ T-cell phenotypes correlated with higher proportion of IT and PT GrB+ NK cells and CD57+ helper T cells.

When studying spatial coordination of immune cells, we observed that higher proportion of PT exhaustion T-cell markers was associated with increased NK cell interaction in the PT region (Supplementary Fig. 11B). However, both IT and PT PD1+TIM3+ and PD1+LAG3+ expression correlated with lower T-cell interaction in the IT region. In summary, these observations suggest that T-cell infiltration and immune checkpoint expression are characterized by distinct spatial and immunophenotypic signatures.

### Immune profiles and cellular interactions associate with clinical variables

**Fig. 4:**
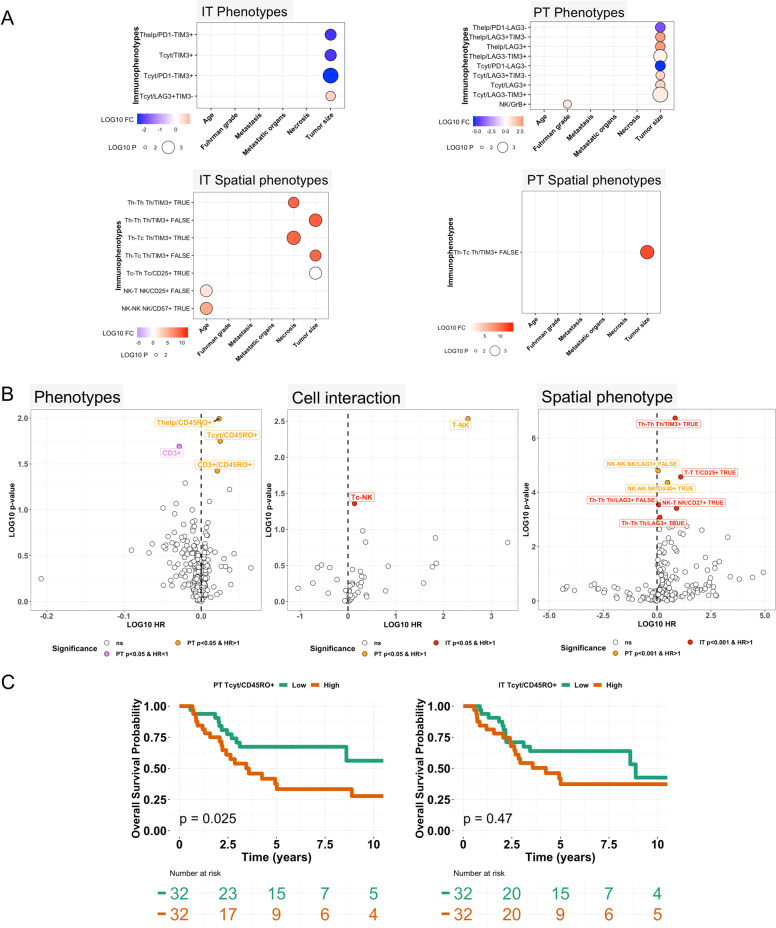
Association of immune cells and clinical variables. **A** Association of clinical factors with IT and PT immunophenotypes (upper) and immunophenotypes based on interaction status (lower). Clinical factors have been categorized into two classes based on median. The color scale represents the log10 fold-change between each subgroup such as high vs. low patient age. Only immunophenotypes significant in any correlation (*p* < 0.05) are shown. **B** IT and PT immunophenotypes, immune cell interaction, and spatial immunophenotypes have been analyzed with Cox regression analysis (log-rank test) for overall survival. **C** Kaplan–Meier visualization of overall survival by IT and PT CD45RO+ cytotoxic T cells.

We hypothesized that immunophenotypes could be linked with clinical factors. Immune cell proportions and location-dependent phenotypes were first examined in the IT region. Increasing tumor size was associated with lower levels of TIM3 expression in T cells, notably when not interacting with other T cells (Fig. 4A). Tumor necrosis was also associated with increased TIM3 expression in interacting helper T cells. In line with previous reports on age-related immune cell dysfunction, CD57 expression was more pronounced in NK cells of elderly patients [40]. Fuhrman grade and the presence or extent of metastasis were not reflected on IT cellular or spatial parameters.

Conversely, we observed distinct associations between tumor extent and immune microenvironment parameters in the PT region. Mixed LAG3+ and TIM3+ immunophenotypes were increased in PT T cells of larger tumors possibly reflecting an immunophenotypic continuum (Fig. 4A). PT GrB+ NK cells were more numerous in tumors characterized with high histological grade. Moreover, elevated TIM3 expression was noted notably in non-interacting helper T cells. However, patient age, metastasis status or necrosis were not reflected on PT spatioimmunological phenotypes.

To further study the clinical significance of T and NK cells and their spatial localization, we investigated their association with overall survival (OS). Memory helper and cytotoxic T cells were enriched in the PT but not IT region of patients with poor OS (Fig. 4B, C). Consistent with the survival benefit reported with excluded immune topography, increased PT CD3+ T-cell proportion predicted also improved prognosis (Fig. 4B). When examining cell interaction patterns, spatial proximity between T and NK cells in both IT and PT regions was associated with poor OS (Fig. 4B). By examining the prognostic impact of immune subtypes in interacting and non-interacting immune cells separately, we found multiple biomarkers of poor OS in the IT region (Fig. 4B). TIM3 and CD25 expression in interacting T cells whereas OX40 and CD27 expression in non-interacting NK cells predicted poor survival. In summary, both spatial dissection of the immunological microenvironment and observation of cell interaction status broadens the potential to discover novel prognostic biomarkers.

## Discussion

In the current study, we applied in situ immunoprofiling coupled with image analysis to study lymphocyte fractions, phenotype and spatial location in IT and PT regions of RCC specimens.

Lymphocyte infiltration was quantitated in whole-slide H&E-stained images to determine immune hot, cold and excluded topographies. The immune topography proportions were validated with TCGA ccRCC digital images. We estimated that our lymphocyte classifier would perform better in cell detection and classification after color normalization possibly as it was developed with a random trees algorithm. However, convolutional neural network-based image analysis could improve classification due to lower sensitivity for technical color variations [41]. Moreover, we found immune excluded tumors to associate with fewer metastatic organs and superior overall survival in our primary dataset, representing a potential clinical biomarker in RCC patients. While this constitutes a promising link between tumor immunology and oncogenic aggressiveness, we note that further validation is needed. TCGA images were not used in this dataset for prognostication due to missing information on systemic treatments.

Recently, Braun et al. [17] investigated the association of immune topographies defined by the spatial quantity of CD8+ cells with anti-PD-1 treatment response in ccRCC patients and found no prognostic impact. However, essential differences in patient cohorts (first-line TKI vs. second-line anti-PD-1), selection of immune cells (lymphocytes vs. CD8+), image analytical approaches and the definition of immune topographies hampers reliable comparison of these studies. The varying results might be due to the lack of guidelines in determining immune topographies. Based on our study and the study of Kather et al. [11], neither the proportion of all immune cell subsets nor the expression of all immunophenotypic markers are elevated in immune hot tumors, suggesting that more robust methods such as CD45 IHC staining should be investigated to define immune topographies. While the unsupervised approach used in this study enabled unbiased immune topography classification in the Helsinki and TCGA ccRCC datasets, independent validation in other tumors is required.

The total mutation burden was not associated with any immune topography. However, we could replicate the association of *PBRM1* mutation and a non-immunogenic tumor phenotype, which has been caused to be related to the downregulation of *IFNγ* target genes [32]. *PBRM1* deficiency has also been associated with poor response to immune checkpoint inhibitors [17, 32]. Here, we demonstrate that altered *STAG2*, which encodes a subunit of the cohesion complex, is associated with an immune hot topography. Moreover, the gene *BAP1* encoding a tumor-suppressor deubiquitinase protein was more frequently deficient in tumors with an immune excluded topography while less frequent for immune hot tumors. Somatic mutations in the *VHL* gene were also less frequent in immune excluded tumors. The analyses did not incorporate methylation or transcriptomic data, which could help to further understand the link between genetic alterations and immune topographies. While these findings require independent validation, these could indicate that individual genes may regulate the organization of tumors into distinct topographies and induce sensitivity or resistance to immunotherapy.

Cellular immunophenotypes were defined with quantitative mIHC. Despite the general interest for immune topographies, separate dissection of IT and PT regions has rarely been conducted, and previously in ccRCC once with a smaller cohort using flow cytometry [42, 43]. Consistent with these results, we showed that the IT region is distinguished by high expression of immune checkpoint receptors in lymphocytes. By studying patient-level immune profiles, we discovered that LAG3 expression in IT T cells drove the unsupervised clustering of RCC patients. Larger renal tumors were associated with reduced IT TIM3+ and more pronounced PT TIM3+ and LAG3+ T cells. PD1 and LAG3 have been reported as the most prevalent T-cell immune checkpoint receptors in RCC, and their blockade to increase IFNγ signaling was considered crucial for successful anti-cancer immunosurveillance [44]. Given the described findings, LAG3+ T cells may represent both a prognostic and therapeutically targeted subset requiring further evaluation. Currently, therapeutic anti-LAG3 antibodies are under clinical investigation for safety and efficacy (NCT01968109, NCT04370704).

HLA-G is highly expressed in placental cytotrophoblasts and at low levels in corneal, endothelial and pancreatic tissues but not in normal kidney tissue [45]. The elevated HLA-G expression observed in RCC patients is consistent with previous publications reporting inferior HLA-G-mediated in vitro immune responses [46]. We did not find associations between HLA-G expression and tumor characteristics. As HLA-G is not expressed in healthy renal tissue, its transcription might be induced due to epigenetic regulation [45]. We reported common expression patterns between HLA-G and PD-L1, which were linked with higher lymphocyte infiltration, and potentially regulation by type I IFN [35, 47]. Therefore, the immunologic targeting of HLA-G may be promising, notably in conjunction with anti-PD1 blockade.

We computed intercellular distances to separately examine interacting and non-interacting cells. While the total proportion of immune cells was lower in the IT region, these were more densely localized and expressed higher levels of immune checkpoint receptors than PT immune cells, in line with the notion that physical proximity is needed for the formation of immunological synapses. T-cell infiltration was characterized with fewer interactions between cytotoxic T cells and CD16+ myeloid cells, elevated PD-L1 and HLA-G expression as well as enrichment of activation-related CD45RO+ and PD1+ lymphocytes. Abundant lymphocyte-lymphocyte and myeloid-myeloid cell interactions, as well as higher PD-L1 and HLA-G expression were observed instead in the context of an immune exhausted profile. In summary, lymphocyte infiltration and immune checkpoint expression are associated with distinct phenotypic compositions.

Comparison of T-cell profiles between IT and PT regions revealed that these represent unique immune contextures. The difference was even more remarkable when examining cellular interaction networks than immune phenotype proportions. The distinct immunophenotypes and interaction indexes may be attributed to the restriction of malignant cells to the IT area and diverse cytokine and stromal microenvironments. Moreover, we identified higher CD45RO expression in interacting compared to non-interacting IT T cells, particularly in conjunction with immune hot tumors. Interestingly, PT CD45RO+ T-cell proportion predicted poor OS suggesting that CD45RO+ T-cell quantity may represent a surrogate marker for T-cell interaction or prognosis depending on the analyzed region in question. We envision that a similar approach combining spatial immunoprofiles with genomic alteration data and immunotherapy treatment response could deepen our understanding of tumor immunology and ccRCC pathology.

We combined whole-slide lymphocyte quantification with tissue microarray-based immune cell immunophenotypes and cellular neighborhoods, highlighting the multifaceted potential of in situ data and spatial analysis. Our study underlines the fundamental priority to dissect both the intratumoral parenchyma and its adjacent peritumoral stroma as these might reflect different immunological profiles and are independently associated with clinicopathologic characteristics. Similar studies based on spatial RNA sequencing will likely emerge. However, collection of large cohorts required to identify biomarkers of aggressive disease or treatment sensitivity might be currently easier to conduct with proteomic methods. In summary, the consideration of tissue contexture and spatial location during tissue sampling is crucial to understand the role of immunosurveillance in cancer.

## Supplementary information

Supplemental material

## Data Availability

Tissue material and image data are available upon reasonable request by contacting the corresponding author. TCGA immune topography classes and lymphocytes proportions are reported in Supplementary data.
