# Supplementary material for: Spatial immunoprofiling of the intratumoral and peritumoral tissue of renal cell carcinoma patients
Source: Mod Pathol. 2021 Jul 2;34(12):2229–41. doi: 10.1038/s41379-021-00864-0 (PMC8592837; doi:10.1038/s41379-021-00864-0)
Supplement: Supplementary file 1 — Supplemental material [file 41379_2021_864_MOESM1_ESM.pdf]

Supplemental material for “Spatial Immunoprofiling of the Intratumoral and Peritumoral Tissue of Renal Cell Carcinoma Patients”

Supplementary Table 1. Immune cell and immune topography proportions in the Helsinki and TCGA ccRCC datasets. As immune cells have been identified with different classifiers, their proportion between datasets are not comparable.

**Dataset**	**TCGA _ccRCC_slide**	**Peritumoral_Immunecells_from_Allcells**	**Intratumoral_Immunecells_from_Allcells**	**Immunetopography**
HKI	Helsinki_1	0.255	0.234	Hot
HKI	Helsinki_2	0.118	0.119	Cold
HKI	Helsinki_3	0.069	0.034	Cold
HKI	Helsinki_4	0.119	0.032	Excluded
HKI	Helsinki_5	0.075	0.098	Cold
HKI	Helsinki_6	0.091	0.043	Cold
HKI	Helsinki_8	0.041	0.047	Cold
HKI	Helsinki_9	0.124	0.099	Cold
HKI	Helsinki_10	0.110	0.218	Hot
HKI	Helsinki_11	0.238	0.176	Hot
HKI	Helsinki_12	0.071	0.083	Cold
HKI	Helsinki_13	0.105	0.071	Cold
HKI	Helsinki_14	0.065	0.041	Cold
HKI	Helsinki_15	0.077	0.040	Cold
HKI	Helsinki_16	0.347	0.131	Hot
HKI	Helsinki_17	0.167	0.063	Excluded
HKI	Helsinki_18	0.215	0.144	Hot
HKI	Helsinki_19	0.108	0.093	Cold
HKI	Helsinki_21	0.218	0.051	Excluded
HKI	Helsinki_22	0.325	0.091	Excluded
HKI	Helsinki_23	0.099	0.114	Cold
HKI	Helsinki_24	0.116	0.136	Hot
HKI	Helsinki_25	0.095	0.097	Cold
HKI	Helsinki_26	0.105	0.041	Cold
HKI	Helsinki_27	0.199	0.106	Excluded
HKI	Helsinki_28	0.076	0.082	Cold
HKI	Helsinki_29	0.163	0.037	Excluded
HKI	Helsinki_30	0.119	0.059	Cold
HKI	Helsinki_31	0.095	0.024	Cold
HKI	Helsinki_33	0.059	0.077	Cold
HKI	Helsinki_34	0.103	0.051	Cold
HKI	Helsinki_37	0.183	0.101	Excluded
HKI	Helsinki_38	0.163	0.043	Excluded
HKI	Helsinki_41	0.142	0.142	Hot

HKI	Helsinki_42	0.178	0.111	Cold
HKI	Helsinki_43	0.167	0.143	Hot
HKI	Helsinki_44	0.269	0.178	Hot
HKI	Helsinki_45	0.191	0.070	Excluded
HKI	Helsinki_46	0.104	0.065	Cold
HKI	Helsinki_47	0.137	0.045	Excluded
HKI	Helsinki_48	0.139	0.075	Cold
HKI	Helsinki_49	0.034	0.097	Cold
HKI	Helsinki_51	0.114	0.068	Cold
HKI	Helsinki_52	0.160	0.155	Hot
HKI	Helsinki_54	0.071	0.065	Cold
HKI	Helsinki_55	0.234	0.190	Hot
HKI	Helsinki_56	0.048	0.018	Cold
HKI	Helsinki_59	0.098	0.011	Excluded
HKI	Helsinki_60	0.212	0.166	Hot
HKI	Helsinki_63	0.111	0.091	Cold
HKI	Helsinki_64	0.763	0.559	Hot
HKI	Helsinki_65	0.043	0.021	Cold
HKI	Helsinki_66	0.105	0.066	Cold
HKI	Helsinki_68	0.173	0.117	Cold
HKI	Helsinki_69	0.120	0.080	Cold
HKI	Helsinki_70	0.007	0.041	Cold
HKI	Helsinki_71	0.043	0.015	Cold
HKI	Helsinki_72	0.032	0.027	Cold
HKI	Helsinki_73	0.101	0.031	Cold
HKI	Helsinki_75	0.107	0.040	Cold
HKI	Helsinki_76	0.205	0.100	Excluded
HKI	Helsinki_77	0.127	0.272	Hot
HKI	Helsinki_78	0.058	0.063	Cold
HKI	Helsinki_79	0.058	0.045	Cold
TCGA	TCGA-3Z-A93Z-01Z-00-DX1_79F4D1A6-ACDB-4AB1-B8A8-C1CEE617C734.svs	0.063	0.089	Hot
TCGA	TCGA-6D-AA2E-01Z-00-DX1_81E7420E-9C8B-4076-A604-F2B2AD3340D5.svs	0.121	0.100	Hot
TCGA	TCGA-A3-3307-01Z-00-DX1_E9BB41BE-9F96-49A7-8164-A7E715A00EB9.svs	0.054	0.029	Cold
TCGA	TCGA-A3-3347-01Z-00-DX1_a06c9ca5-97d5-46a8-95e5-7bf9570a6dc7.svs	0.104	0.033	Excluded
TCGA	TCGA-A3-3359-01Z-00-DX1_D473A2D2-AE66-469D-80A1-584598335E17.svs	0.227	0.093	Hot
TCGA	TCGA-AK-3458-01Z-00-DX1_45890D8B-B66A-49C9-983A-4A2AD6722076.svs	0.052	0.029	Cold

TCGA	TCGA-B0-4697-01Z-00-DX1_f897b21b-75c7-4ebc-86d9-8d783b39fefa.svs	0.024	0.020	Cold
TCGA	TCGA-B0-4699-01Z-00-DX1_18b6d6e7-6e37-4bcd-922c-ce81e2e81067.svs	0.081	0.050	Cold
TCGA	TCGA-B0-4706-01Z-00-DX1_a29630d2-be74-4245-8d47-2e1694bea5e4.svs	0.023	0.039	Cold
TCGA	TCGA-B0-4707-01Z-00-DX1_861325d7-cd42-4564-87f7-53deccc410d8.svs	0.038	0.036	Cold
TCGA	TCGA-B0-4834-01Z-00-DX1_c4bb0354-50a6-4ac7-b538-59a9428bc527.svs	0.028	0.032	Cold
TCGA	TCGA-B0-4839-01Z-00-DX1_0c13b082-d7e6-4327-b5cc-ab6bd99b78aa.svs	0.124	0.090	Hot
TCGA	TCGA-B0-4841-01Z-00-DX1_a33f52a7-d045-4aaa-acbe-e3122f33147c.svs	0.095	0.100	Hot
TCGA	TCGA-B0-4842-01Z-00-DX1_d780158b-81c2-4ac9-b7a0-9c9386c6414c.svs	0.106	0.109	Hot
TCGA	TCGA-B0-4849-01Z-00-DX1_979ac1bc-d04f-470d-bed2-85d3bd3cd912.svs	0.032	0.019	Cold
TCGA	TCGA-B0-4945-01Z-00-DX1_590b650c-c9cb-4601-886c-fde0ccd9b90d.svs	0.064	0.021	Excluded
TCGA	TCGA-B0-5081-01Z-00-DX1_2fed8120-8e9c-4a23-bd1b-645f91450464.svs	0.070	0.081	Hot
TCGA	TCGA-B0-5085-01Z-00-DX1_29c82ff0-9e4d-477f-a8d9-8931c69e58f8.svs	0.027	0.056	Cold
TCGA	TCGA-B0-5098-01Z-00-DX1_d9298d58-2fd2-4cfd-9900-6e0b7ebfc9c9.svs	0.235	0.062	Excluded
TCGA	TCGA-B0-5099-01Z-00-DX1_b52a09b0-7361-4bc8-afce-d587d716599a.svs	0.043	0.041	Cold
TCGA	TCGA-B0-5100-01Z-00-DX1_52baec4e-41bf-4ddc-ac0d-8058f8078018.svs	0.067	0.104	Hot
TCGA	TCGA-B0-5102-01Z-00-DX1_0dc0a4bf-4836-4f9f-a31e-c03eb6855cf7.svs	0.045	0.069	Cold
TCGA	TCGA-B0-5106-01Z-00-DX1_564e51a3-be50-4eed-ba9c-4d478b96ffc6.svs	0.045	0.062	Cold
TCGA	TCGA-B0-5115-01Z-00-DX1_f07e128b-5a67-409a-a420-ce953f291364.svs	0.076	0.084	Hot

TCGA	TCGA-B0-5117-01Z-00-DX1_d847a765-e0f0-4dfe-89e6-d85a1145e3ad.svs	0.116	0.049	Excluded
TCGA	TCGA-B0-5119-01Z-00-DX1_0efec19e-0a46-4951-926b-d172fb90b2a1.svs	0.010	0.028	Cold
TCGA	TCGA-B0-5690-01Z-00-DX1_3f15b28e-03d0-43e9-b289-2697316f4e30.svs	0.089	0.068	Cold
TCGA	TCGA-B0-5691-01Z-00-DX1_ef332029-fdef-4a0c-b891-4ef14da0e6ba.svs	0.018	0.044	Cold
TCGA	TCGA-B0-5695-01Z-00-DX1_60ef3025-1240-4d7d-b73f-28b27fa094d8.svs	0.133	0.091	Hot
TCGA	TCGA-B0-5696-01Z-00-DX1_73c27995-9401-43bd-80ff-6ee9dadcbd5a.svs	0.029	0.038	Cold
TCGA	TCGA-B0-5699-01Z-00-DX1_e53ec6c8-f6c1-4669-bcdd-c2d04acc6a08.svs	0.031	0.034	Cold
TCGA	TCGA-B0-5702-01Z-00-DX1_a455181a-c4a5-40f5-b82e-be5bc6bb0a41.svs	0.139	0.133	Hot
TCGA	TCGA-B0-5703-01Z-00-DX1_c358bcd6-0d76-459d-862d-041ab3ebb496.svs	0.179	0.114	Hot
TCGA	TCGA-B0-5706-01Z-00-DX1_3e5b4737-48e5-44af-b029-f4091812c13b.svs	0.033	0.040	Cold
TCGA	TCGA-B0-5709-01Z-00-DX1_8e4b701a-1ef7-4d79-a562-0f316c696984.svs	0.168	0.093	Hot
TCGA	TCGA-B0-5711-01Z-00-DX1_6839921f-ec08-4e94-8bf9-4b5a4fcdda45.svs	0.023	0.079	Hot
TCGA	TCGA-B0-5812-01Z-00-DX1_668e75ce-2d02-47aa-9ce4-9931dd53e9a4.svs	0.024	0.034	Cold
TCGA	TCGA-B4-5843-01Z-00-DX1_6e020ad3-b4fc-4280-ab98-865eb075fe29.svs	0.079	0.071	Cold
TCGA	TCGA-BP-4159-01Z-00-DX1_e3affffd-b9fa-4874-8c2f-427458d18020.svs	0.112	0.086	Hot
TCGA	TCGA-BP-4160-01Z-00-DX1_3d2b326c-6bab-46b6-999f-c9e244524926.svs	0.066	0.104	Hot
TCGA	TCGA-BP-4163-01Z-00-DX1_1dc1c4fb-2691-42f8-b62f-c51db47b30dc.svs	0.121	0.069	Excluded
TCGA	TCGA-BP-4164-01Z-00-DX1_9834000b-0e64-46b0-ad7f-9c18b93bf4c0.svs	0.059	0.068	Cold

TCGA	TCGA-BP-4325-01Z-00-DX1_f3299377-fdc2-4ac0-b26e-487ef77cac8b.svs	0.130	0.098	Hot
TCGA	TCGA-BP-4327-01Z-00-DX1_fd80b9b2-ec68-4e6a-b0ce-a08c9baba9b7.svs	0.158	0.077	Hot
TCGA	TCGA-BP-4329-01Z-00-DX1_da54fc65-7cb6-4265-8d45-b373a59fa1da.svs	0.189	0.061	Excluded
TCGA	TCGA-BP-4331-01Z-00-DX1_59a3b421-c402-451e-ad82-dda54583d3d4.svs	0.061	0.091	Hot
TCGA	TCGA-BP-4338-01Z-00-DX1_fbadb1d7-c11d-4347-9f3c-447f52dde639.svs	0.197	0.098	Hot
TCGA	TCGA-BP-4343-01Z-00-DX1_262f0e99-f404-4232-bd7a-b23f2ebefc49.svs	0.130	0.041	Excluded
TCGA	TCGA-BP-4344-01Z-00-DX1_a6ed5255-894e-494c-83bd-736daedd1d5c.svs	0.076	0.049	Cold
TCGA	TCGA-BP-4758-01Z-00-DX1_f30694f7-b54f-43d8-a71c-0558caf0621c.svs	0.114	0.111	Hot
TCGA	TCGA-BP-4760-01Z-00-DX1_0a49c368-739d-42f8-a1cc-5a35ee70a810.svs	0.066	0.037	Cold
TCGA	TCGA-BP-4769-01Z-00-DX1_c13d841b-b3f6-4d86-b1c1-94e850a8fa44.svs	0.070	0.055	Cold
TCGA	TCGA-BP-4777-01Z-00-DX1_d974c34b-6e2e-419e-8d4e-674380f313ba.svs	0.067	0.022	Excluded
TCGA	TCGA-BP-4787-01Z-00-DX1_8bac7e59-04be-43a6-8656-501bf157452c.svs	0.125	0.048	Excluded
TCGA	TCGA-BP-4797-01Z-00-DX1_ede57976-a7d8-4534-bba3-3a3472fefd62.svs	0.091	0.101	Hot
TCGA	TCGA-BP-4803-01Z-00-DX1_6d00ac00-b812-44bf-a826-b24194bd8ab0.svs	0.125	0.068	Excluded
TCGA	TCGA-BP-4804-01Z-00-DX1_a998cc6f-408c-4144-9d8f-6a800bb85ced.svs	0.104	0.041	Excluded
TCGA	TCGA-BP-4959-01Z-00-DX1_2f8b4ccb-c280-48e3-b901-50ef83e56669.svs	0.080	0.035	Excluded
TCGA	TCGA-BP-4960-01Z-00-DX1_8ed78985-d391-4756-ab30-eca545091013.svs	0.049	0.021	Cold
TCGA	TCGA-BP-4962-01Z-00-DX1_49d7d740-c260-467d-8ce2-3bf4e6fe6ef0.svs	0.114	0.062	Excluded

TCGA	TCGA-BP-4965-01Z-00-DX1_80afb4f3-e44e-4e99-8047-102be4aad2de.svs	0.186	0.054	Excluded
TCGA	TCGA-BP-4974-01Z-00-DX1_97991078-9139-4bca-a5c7-0c6c66459df9.svs	0.148	0.087	Hot
TCGA	TCGA-BP-4975-01Z-00-DX1_3b4b3a5f-f2c1-41d7-9590-11dc5a6265d9.svs	0.080	0.089	Hot
TCGA	TCGA-BP-4985-01Z-00-DX1_46854f1c-2f91-4990-b0ca-c76ff09bd835.svs	0.082	0.064	Cold
TCGA	TCGA-BP-4988-01Z-00-DX1_b037f17b-8fc7-46b2-adae-8e1140606fb7.svs	0.063	0.053	Cold
TCGA	TCGA-BP-4992-01Z-00-DX1_3f676d40-00de-4eb4-971d-6b93ed165cf5.svs	0.160	0.121	Hot
TCGA	TCGA-BP-4993-01Z-00-DX1_9eb12eba-9015-4f39-a241-8038ab4ae4a7.svs	0.036	0.032	Cold
TCGA	TCGA-BP-4995-01Z-00-DX1_e8ebf283-46f9-4d1f-a8a6-11c24d8fad46.svs	0.023	0.024	Cold
TCGA	TCGA-BP-4998-01Z-00-DX1_f3ea98d5-3807-47d3-bcd6-5501d7679b88.svs	0.043	0.045	Cold
TCGA	TCGA-BP-5001-01Z-00-DX1_05212dc9-c0cf-4fbb-96c3-f39f6ed67ca4.svs	0.064	0.069	Cold
TCGA	TCGA-BP-5007-01Z-00-DX1_3d9c2588-3310-4fbc-8bc0-7185e9b0d83b.svs	0.096	0.076	Hot
TCGA	TCGA-BP-5009-01Z-00-DX1_54e2bdc1-1010-4f1f-9eea-e4a7e2c12341.svs	0.116	0.079	Hot
TCGA	TCGA-BP-5174-01Z-00-DX1_d6f8427f-fbbe-4582-a020-ede5675a7e76.svs	0.040	0.058	Cold
TCGA	TCGA-BP-5175-01Z-00-DX1_e954ae94-307c-475e-9f63-92ae1fe946d5.svs	0.176	0.110	Hot
TCGA	TCGA-BP-5178-01Z-00-DX1_4d59b151-8cc6-451a-b009-a4f3a46ae653.svs	0.144	0.069	Excluded
TCGA	TCGA-BP-5180-01Z-00-DX1_accba421-72e5-41f9-8830-ef4aa84e79ec.svs	0.045	0.038	Cold
TCGA	TCGA-BP-5181-01Z-00-DX1_cc8df798-7f23-43a8-abb4-96e84afa2271.svs	0.101	0.093	Hot
TCGA	TCGA-BP-5183-01Z-00-DX1_f84b4982-b51e-4220-a80b-767ecbb3e20f.svs	0.048	0.051	Cold

TCGA	TCGA-BP-5185-01Z-00-DX1_e965a765-7610-4299-8a21-b4e004f6892e.svs	0.098	0.109	Hot
TCGA	TCGA-BP-5186-01Z-00-DX1_d4aa0ed3-0eb7-4317-ba06-decced7e57f1.svs	0.047	0.031	Cold
TCGA	TCGA-BP-5190-01Z-00-DX1_1bc4a76e-f264-45fc-ad34-6c77593c4cc4.svs	0.064	0.046	Cold
TCGA	TCGA-BP-5191-01Z-00-DX1_4942ebf2-cef3-4cff-8430-b377d274952f.svs	0.160	0.135	Hot
TCGA	TCGA-BP-5196-01Z-00-DX1_85bc950b-ed4a-4f77-8c17-b2916d0a05de.svs	0.129	0.058	Excluded
TCGA	TCGA-BP-5198-01Z-00-DX1_3ff31173-5a84-4a07-92cf-2dec884f3c64.svs	0.038	0.018	Cold
TCGA	TCGA-CJ-4640-01Z-00-DX1_F5625E6F-1B5D-408A-982B-9A66103DF1CC.svs	0.027	0.042	Cold
TCGA	TCGA-CJ-4641-01Z-00-DX1_887252E8-C300-4DB2-A6D2-BA47AC6EFBB2.svs	0.136	0.065	Excluded
TCGA	TCGA-CJ-4642-01Z-00-DX1_C21B27E4-341C-471E-8CEE-820348D4F24A.svs	0.112	0.126	Hot
TCGA	TCGA-CJ-4643-01Z-00-DX1_50F38125-1825-4B66-A171-4C92279E306D.svs	0.067	0.061	Cold
TCGA	TCGA-CJ-4868-01Z-00-DX1_95E8398F-7709-42F0-9F2B-2D185B4429AE.svs	0.138	0.050	Excluded
TCGA	TCGA-CJ-4876-01Z-00-DX1_CE682056-925D-456B-AD68-53975CC082D5.svs	0.119	0.088	Hot
TCGA	TCGA-CJ-4878-01Z-00-DX1_33EAAB31-7D61-4479-B590-C354B9256433.svs	0.052	0.031	Cold
TCGA	TCGA-CJ-4885-01Z-00-DX1_7B74D046-B821-485E-934E-6D3278355822.svs	0.019	0.025	Cold
TCGA	TCGA-CJ-4897-01Z-00-DX1_7C8DAACB-B64D-4F7C-B543-B2C19D041F51.svs	0.057	0.038	Cold
TCGA	TCGA-CJ-4902-01Z-00-DX1_5A7985BA-A46C-4769-8C99-9CCCB3A5EDC8.svs	0.271	0.091	Hot
TCGA	TCGA-CJ-4903-01Z-00-DX1_6DB22AEA-582B-44CB-B153-CBBFE5042735.svs	0.081	0.060	Cold

TCGA	TCGA-CJ-4905-01Z-00-DX1_D9E4CA77-5766-43EA-83BA-1C70E28661C1.svs	0.019	0.020	Cold
TCGA	TCGA-CJ-4908-01Z-00-DX1_3B9E62AC-B67F-4AAC-9255-8A1EB3A78473.svs	0.068	0.034	Cold
TCGA	TCGA-CJ-4916-01Z-00-DX1_9C306902-51B1-4968-BB06-DE21606DD963.svs	0.103	0.078	Hot
TCGA	TCGA-CJ-6030-01Z-00-DX1_A762AB76-62E5-4680-9912-8B6D7FC48FF9.svs	0.209	0.108	Hot
TCGA	TCGA-CJ-6032-01Z-00-DX1_37FA1176-310F-4A01-B944-501129FB797D.svs	0.097	0.043	Excluded
TCGA	TCGA-CJ-6033-01Z-00-DX1_5DD2C8F4-50E1-47C3-B5D4-058AE04095DD.svs	0.134	0.032	Excluded
TCGA	TCGA-CZ-4859-01Z-00-DX1_ef46368c-c76e-4fc9-a620-65b7f63fb083.svs	0.064	0.092	Hot
TCGA	TCGA-CZ-4865-01Z-00-DX1_54659e9f-398c-45d9-93c9-35a75ca62509.svs	0.034	0.061	Cold
TCGA	TCGA-CZ-5458-01Z-00-DX1_56cfd26c-44c1-4971-a59b-443742fd78a8.svs	0.352	0.143	Hot
TCGA	TCGA-CZ-5464-01Z-00-DX1_093c64f0-b49e-48a8-a3af-ed0af4b6a548.svs	0.219	0.113	Hot
TCGA	TCGA-CZ-5466-01Z-00-DX1_f0fd74a8-2430-45dd-9c76-8794619ccf9d.svs	0.238	0.125	Hot
TCGA	TCGA-CZ-5468-01Z-00-DX1_e2bbe417-a24c-4511-934e-674221855695.svs	0.109	0.069	Excluded
TCGA	TCGA-CZ-5989-01Z-00-DX1_c6fd2257-4742-4c4e-ade2-995f54f1cfa8.svs	0.116	0.058	Excluded
TCGA	TCGA-DV-5566-01Z-00-DX1_715a6d3c-1172-4f06-8077-32d2a4efb5b3.svs	0.062	0.065	Cold
TCGA	TCGA-DV-5569-01Z-00-DX1_6ac6fcb6-18df-4838-83eb-d339f8ce0722.svs	0.016	0.019	Cold
TCGA	TCGA-DV-5574-01Z-00-DX1_29e43c01-6f4b-4e06-8097-bbe69a187de0.svs	0.100	0.115	Hot
TCGA	TCGA-DV-5575-01Z-00-DX1_2f0a5606-1df8-4c3d-aba5-60f3b7d023e5.svs	0.045	0.038	Cold
TCGA	TCGA-DV-A4W0-01Z-00-DX1_B9EFDA21-A24B-46B2-999A-9E0865615D27.svs	0.109	0.064	Excluded

Supplementary Table 2. Antibodies used in multiplex IHC

Marker	Manufacturer	Clone	Host species
CD2	Cell Marque	MRQ-11	Mouse
CD3	Thermo Scientific	EP449E	Rabbit
CD4	Abcam	EPR6855	Rabbit
CD8	BioSB	C8/144B	Mouse
CD16	LsBio	SP175	Rabbit
CD25	Abcam	EPR6452	Rabbit
CD27	Sigma	Polyclonal	Rabbit
CD45RO	Abcam	UCH-L1	Mouse
CD57	Sigma	VC1.1	Mouse
PD1	LsBio	PDCD1	Mouse
TIM3	Cell Signaling	D5D5R	Rabbit
LAG3	LsBio	LS-C340097	Mouse
OX40	Biolegend	ACT35	Mouse
Granzyme B	Novocastra	11F1	Mouse
PDL1	Cell Signaling	E1L3N	Rabbit
HLA ABC	MBL International Corporation	EMR8-5	Mouse
HLA G	Santa Cruz	4H84	Mouse
ECadH	BD	36/E	Mouse
Pan-Cytokeratin	Thermo Scientific	AE 1/3	Mouse
Pan-Cytokeratin	Abcam	C-11	Mouse
CAIX	Novus	NB100417	Rabbit
AMACR	Abcam	2A10F3	Mouse
CK5	Abcam	2C2	Mouse

Supplementary Table 3. Multiplex IHC antibody panels and antibody dilutions. Abbreviations: LPR, liquid permanent red; VG, vina green; GrB, granzyme B. RCC marker*: ECadH 1:5000, AE 1/3 1:1000, C-11 1:1500, CAIX (1:3000), AMACR (1:2000), CK5 (1:5000).

**Panels/Detection probes**	**GFP**	**Cy3**	**Cy5**	**Cy7**	**LPR**	**VG**
**Cells**	CD16 1:500	CD2 1:500	CD8 1:25	CD4 1:25	CD3 1:250	CD45RO 1:150
**T-cell checkpoints**	LAG3 1:1750	CD3 1:500	CD8 1:25	CD4 1:25	PD1 1:500	TIM-3 1:75
**NK-cell checkpoints**	LAG3 1:1750	OX40 1:25	CD2 1:25	CD3 1:250	PD1 1:500	TIM-3 1:75
**T-cell activity**	GrB 1:100	CD25 1:25	CD8 1:25	CD4 1:25	CD3 1:250	CD57 1:200
**NK-cell activity**	GrB 1:100	CD25 1:25	CD2 1:25	CD27 1:25	CD3 1:250	CD57 1:200
**Cancer immune ligands**	HLA G 1:25	PD-L1 1:50		HLA ABC 1:100	RCC marker*	

Supplementary Table 4. Cell classification with multiplex IHC

**Cell class**	**Marker combination**
NK cell	CD2+CD3-
T cell	CD3+
CD16+ Myeloid cell	CD16+
Helper T cell	CD3+CD4+
Cytotoxic T cell	CD3+CD8+

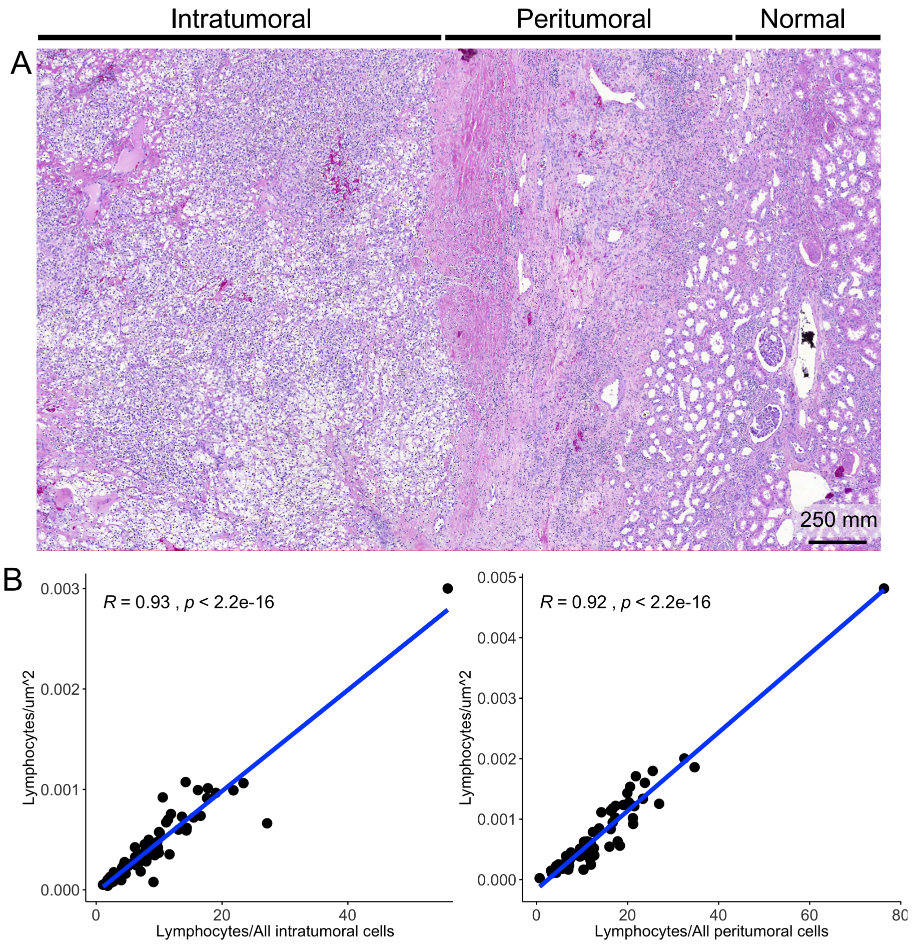

Supplementary Figure 1. (A) Representative H&E staining of nephrectomy-operated renal cell carcinoma tissue with distinct intratumoral (IT), peritumoral (PT) and normal healthy tissue. (B) Linear regression line and Spearman correlation (left upper corner) with IT (left figure) and PT (right figure) lymphocyte proportion from all cells (x-axis) and from analyzed tissue area (y-axis).

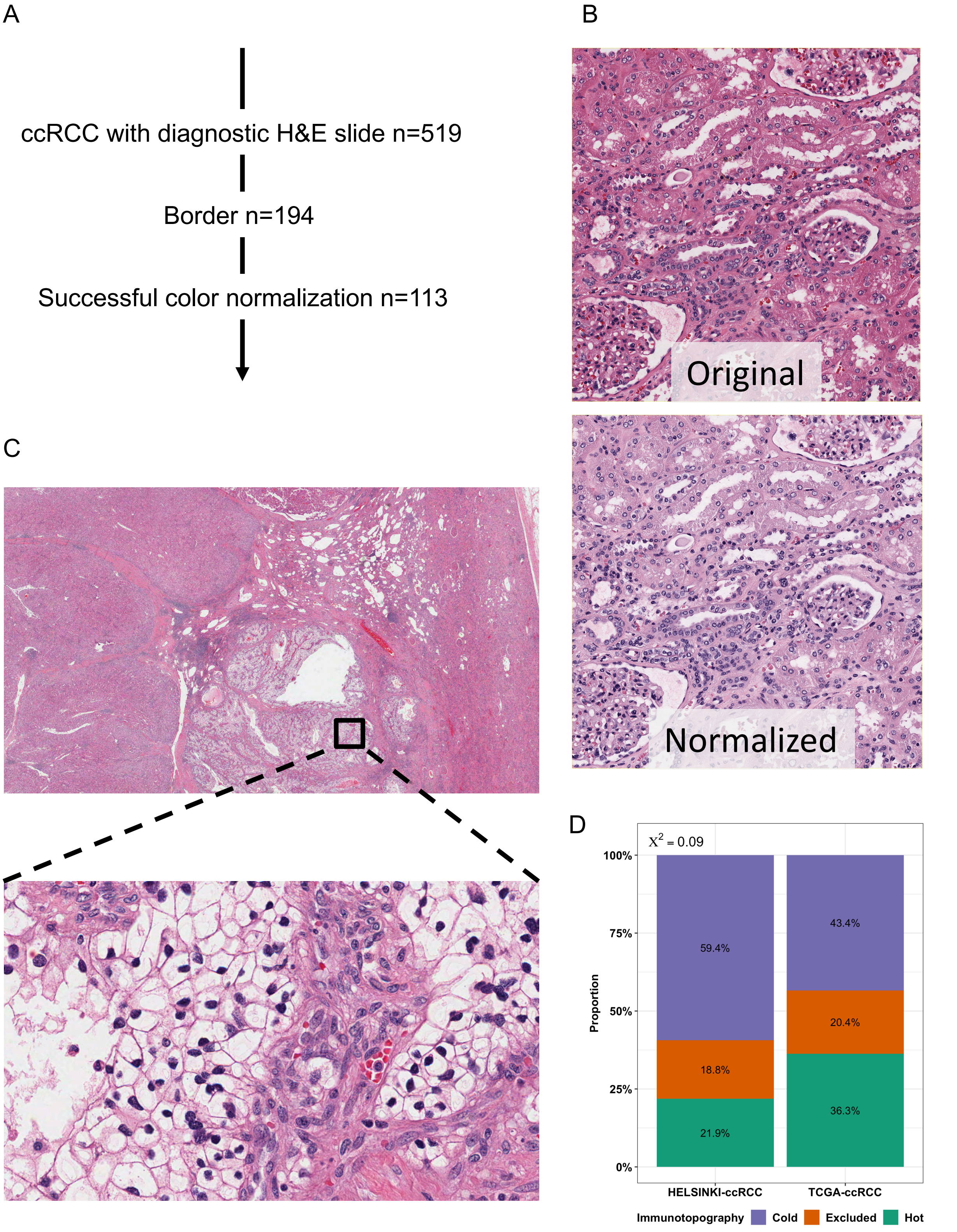

Supplementary Figure 2. Color normalization of The Cancer Genome Atlas (TCGA) digital slide repository. (A) From all H&E stained clear-cell renal cell carcinoma diagnostic slides, we identified 194 samples with representative intratumoral and peritumoral tissue. (B) These images were color normalized with a structure-preserving color normalization method (C) by using the TCGA-B0-4691-01Z-00-DX1 slide as reference image. (D) Lymphocytes were detected from all cells and their proportions in intratumoral and peritumoral regions classified into immune topographies. These were compared between the Helsinki and the TCGA cohorts with the Chi^2 test.

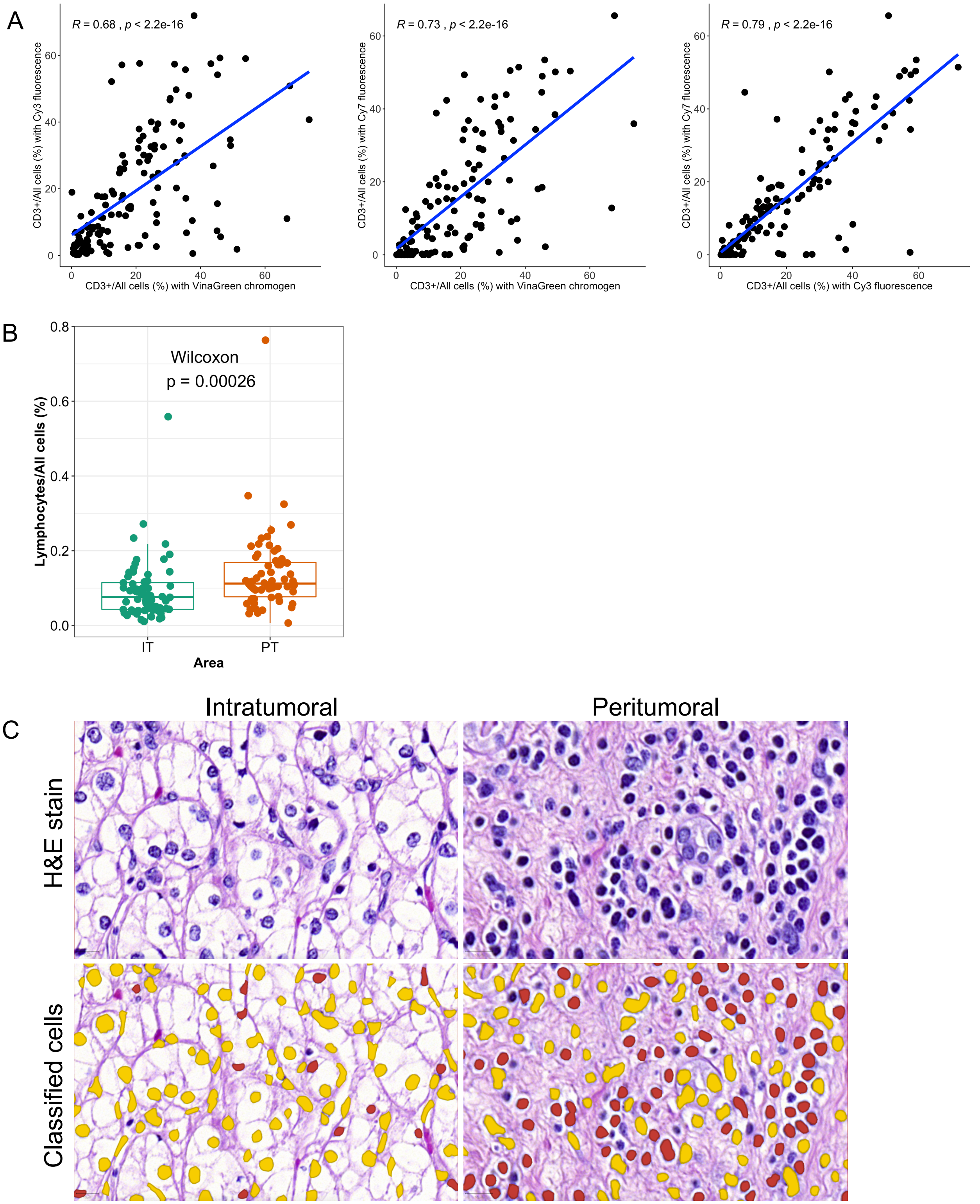

Supplementary Figure 3. (A) Linear regression line and Spearman correlation (left upper corner) of CD3+ cell proportion quantitated with VinaGreen chromogen detection (“Cells” panel), Cy3 fluorescence channel (“T-cell Checkpoints”) and Cy7 fluorescence channel (“NK-cell Checkpoints”). CD3+ proportions have been stained from three sequencial slides. CD3+ proportions from both intratumoral and peritumoral tissue regions are included as separate observations. High correlation reflects consintency across both separate tissue sections and detection methods. (B) Lymphocyte proportions have been compared by the tissue origin with Wilcoxon test. (C) Result of cell classification (bottom panel) from H&E stained tissue (upper panel). Lymphocytes (red) have been identified from other cells (yellow).

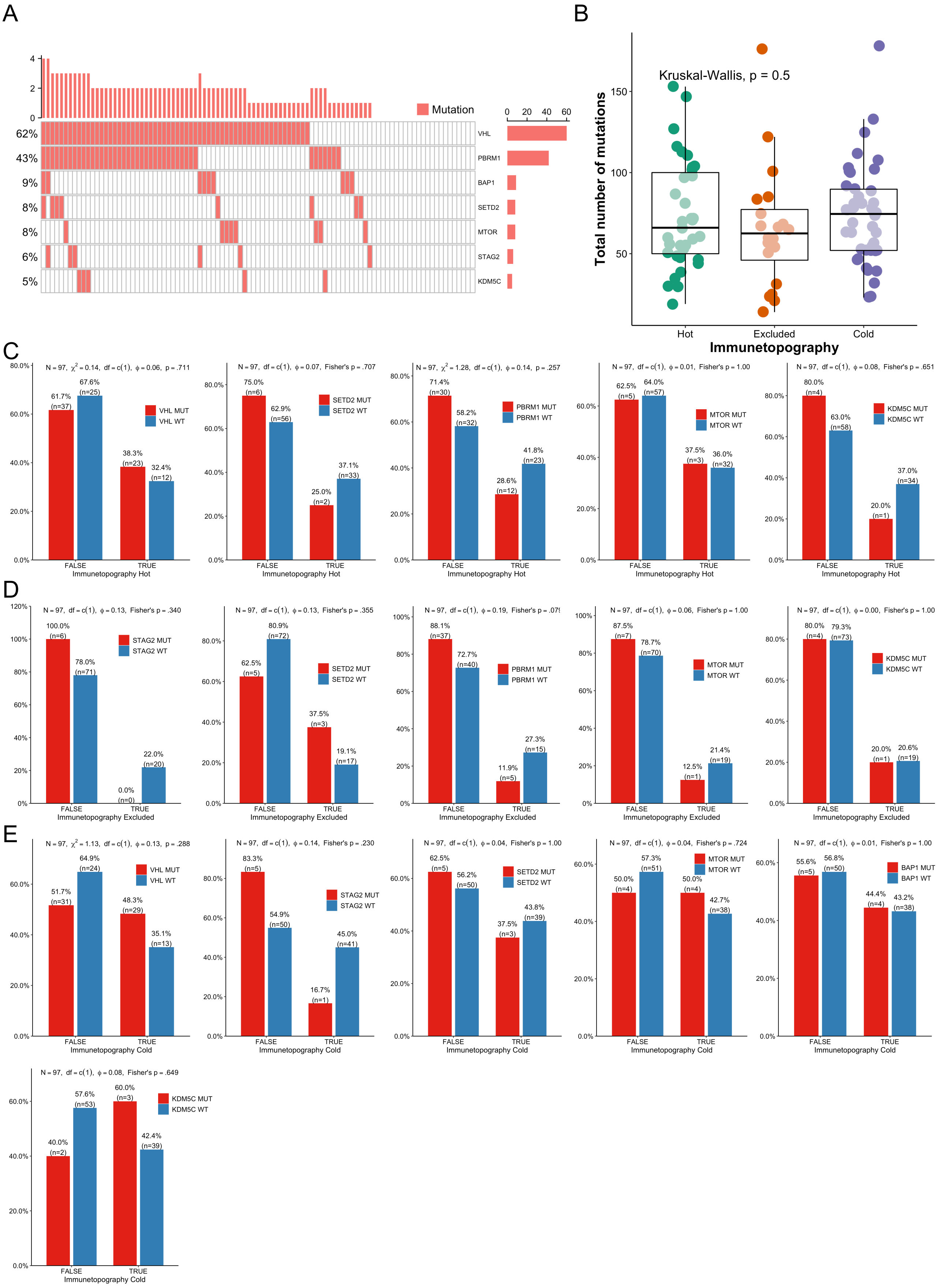

Supplementary Figure 4. Association of genetic alterations and immune topographies in The Cancer Genome Atlas dataset. (A) An oncoprint of somatic alterations by rows and patients by columns. (B) Scatter and box plot of the total number of mutations and immune topographies (Wilcoxon test). (C) Barplots of genetic alterations in most commonly mutated genes and tumors with an immune hot, (D) immune excluded, and (E) immune cold topography.

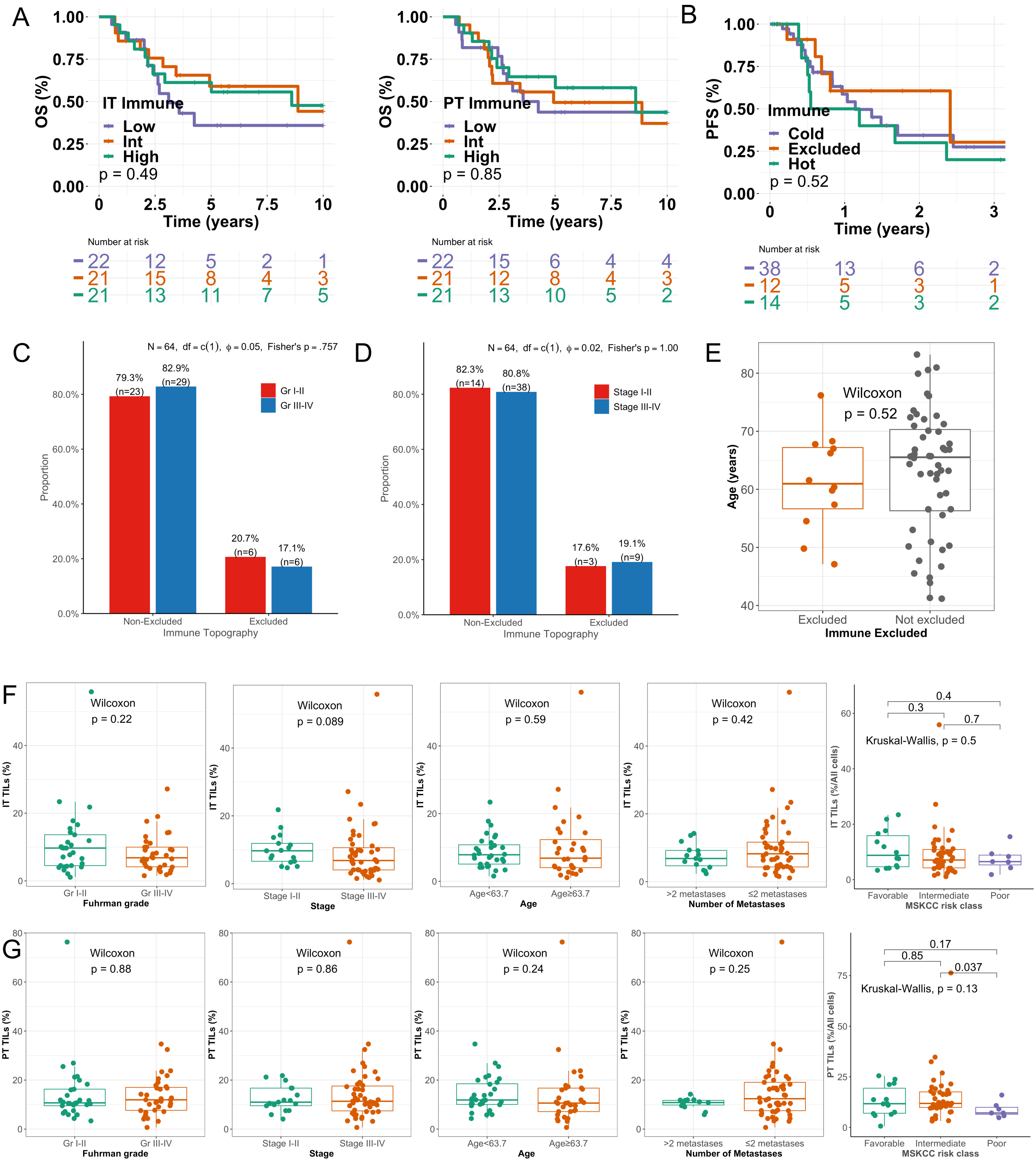

Supplementary Figure 5. (A) Kaplan-Meier (log-rank test) visualization of overall survival by intratumoral (IT) lymphocyte proportion, and peritumoral (PT) lymphocyte proportion. (B) Kaplan-Meier (log-rank test) visualization of progression-free survival (PFS) by immunotopographies. (C) Barplot of the distribution of histological grade and (D) tumor stage by excluded immune topography (Fisher's test). (E) Scatter and box plot of patient age by excluded immune topography (Wilcoxon test). (F) Scatter and box plot of IT and (G) PT tumor-infiltrating lymphocytes (TILs) by Fuhrman grade, tumor stage, patient age, number of metastases and MSKCC risk score (Wilcoxon test).

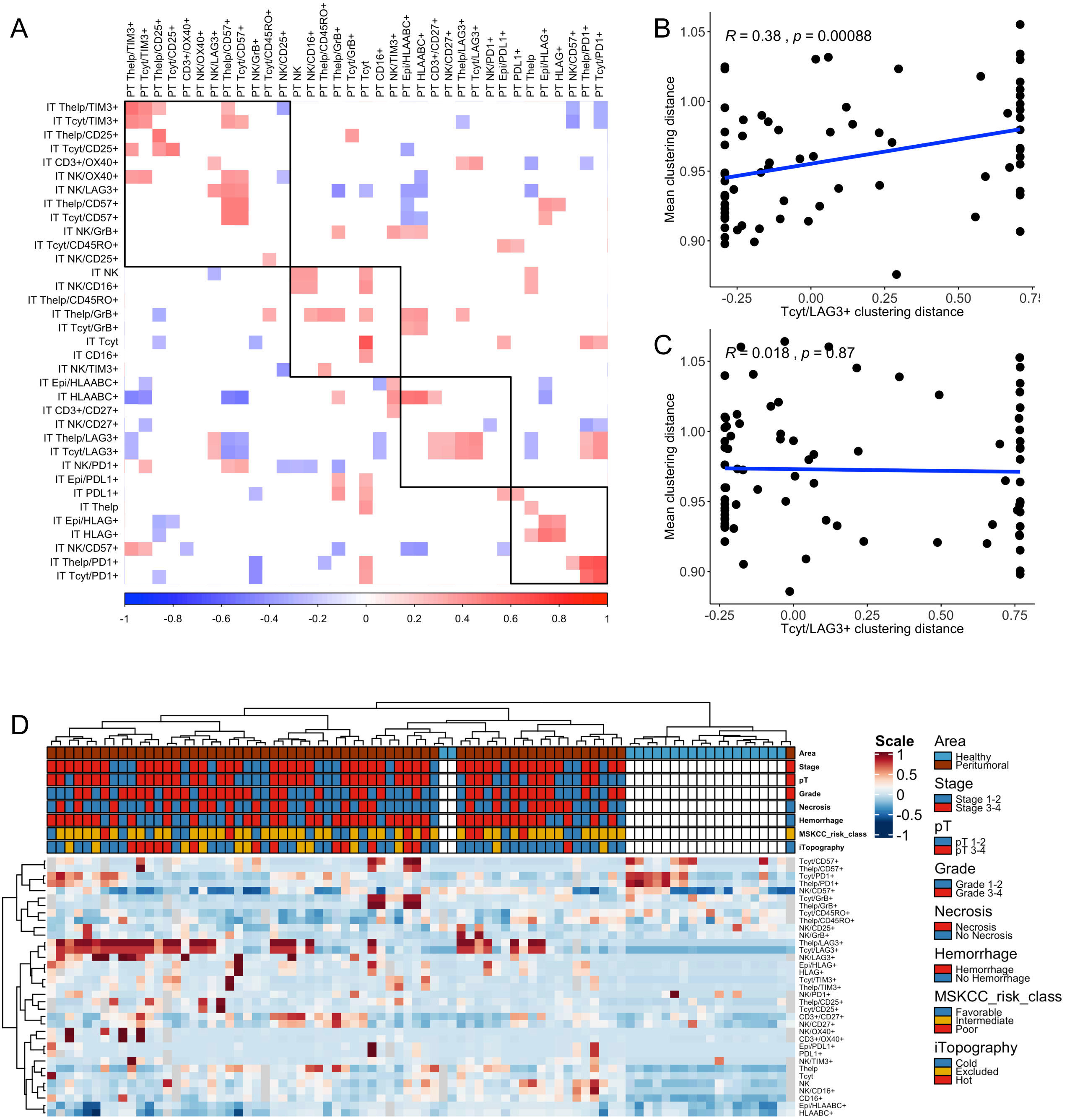

Supplementary Figure 6. (A) Spearman correlation matrix of intratumoral (IT) and peritumoral (PT) immunophenotypes. P-values have been adjusted with Benjamin & Hochberg correction. Only significant correlations (Benjamini&Hochberg adjusted p-value <0.05 are color-labelled). (B) Spearman correlation of the hierarchical clustering of the mean clustering distance with the proportion of IT LAG3+ cytotoxic T cells and (C) with the proportion of PT LAG3+ cytotoxic T cells. The clustering distance has been defined as the 1 - correlation coefficient (Spearman correlation) of heatmaps in Figure 2e and Supplementary Figure 3d. (D) Patient-level heatmap of immunophenotypes in PT and control tissues. Hierarchical clustering has been computed using Spearman correlation as distance.

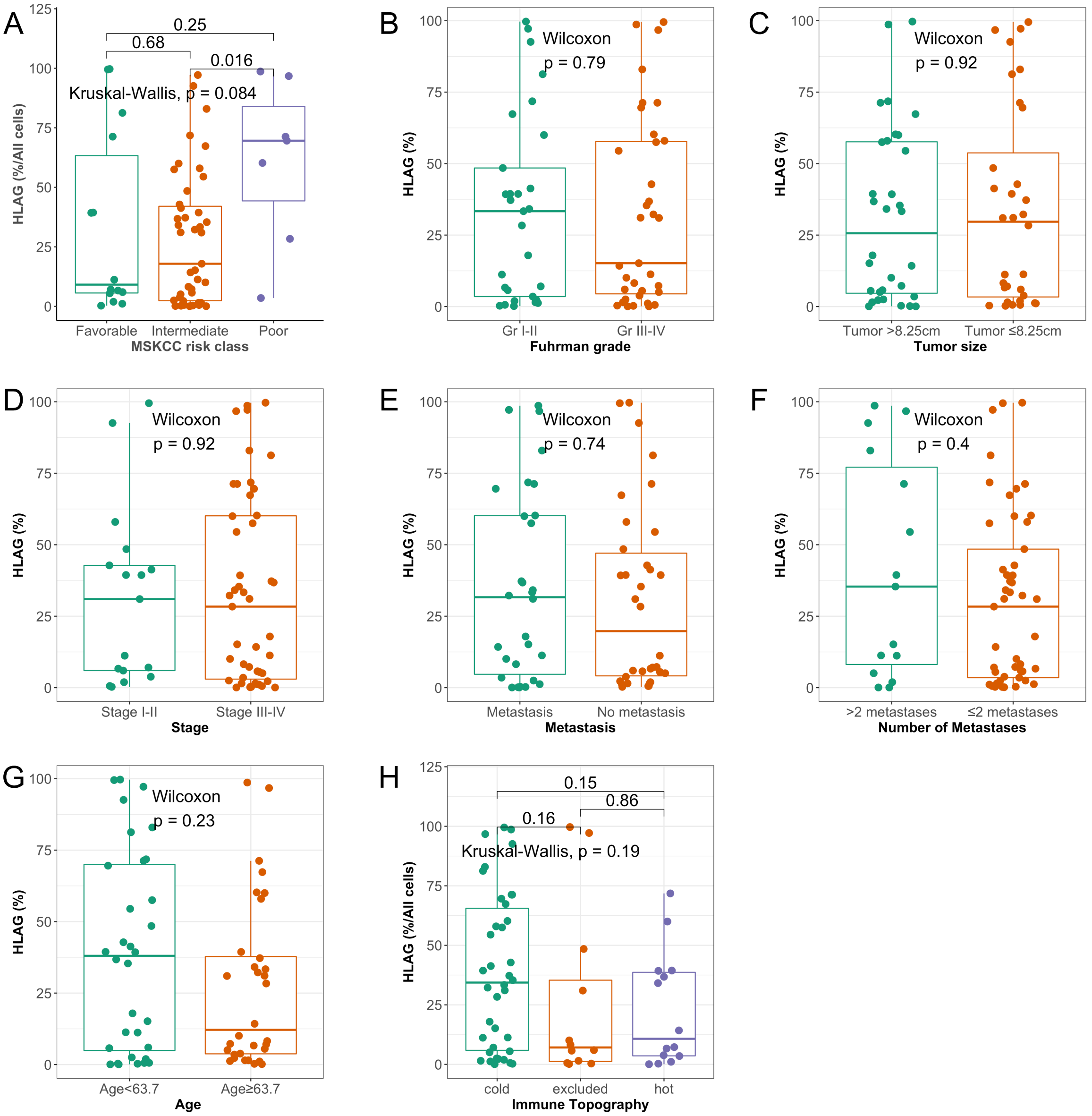

Supplementary Figure 7. (A) Scatter and box plots for the comparison of HLA-G levels defined with multiplex immunohistochemistry and MSKCC risk class, (B) tumor Fuhrman grade, (C) tumor size, (D) tumor stage, (E) metastasis status at diagnosis, (F) number of metastatic organs, (G) patient age and (H) immune topography.

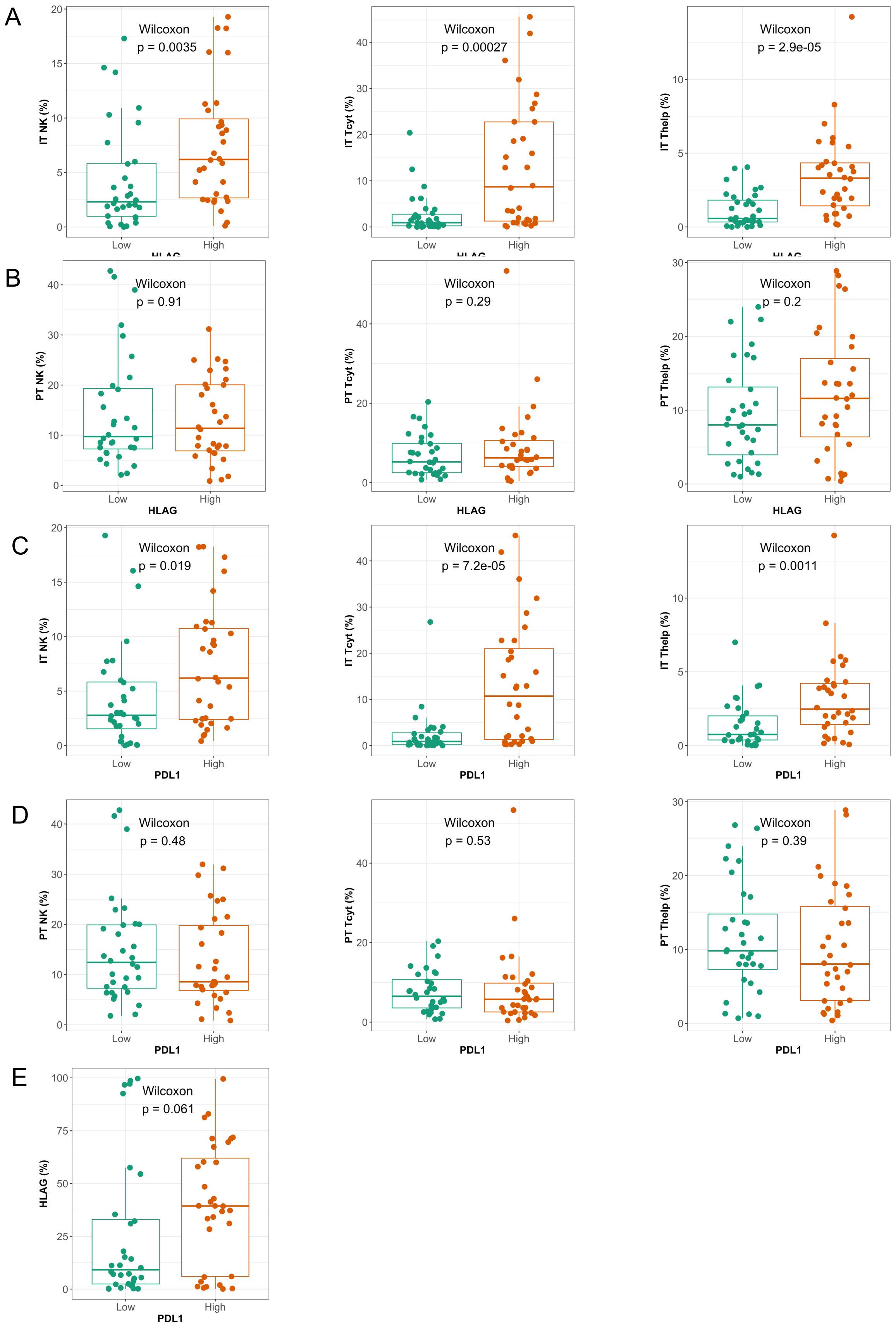

Supplementary Figure 8. (A) Scatter and box plots for the comparison of HLA-G levels defined with multiplex immunohistochemistry and NK, helper and cytotoxic T cells in the intratumoral (IT) and (B) peritumoral (PT) region. (C) Scatter plots for the comparison of PD-L1 levels defined with immunohistochemistry and NK, helper and cytotoxic T cells in the IT and (D) PT region. (E) Scatter plot for the comparison of HLA-G levels by PD-L1 levels. PD-L1 categorized have been defined with median expression.

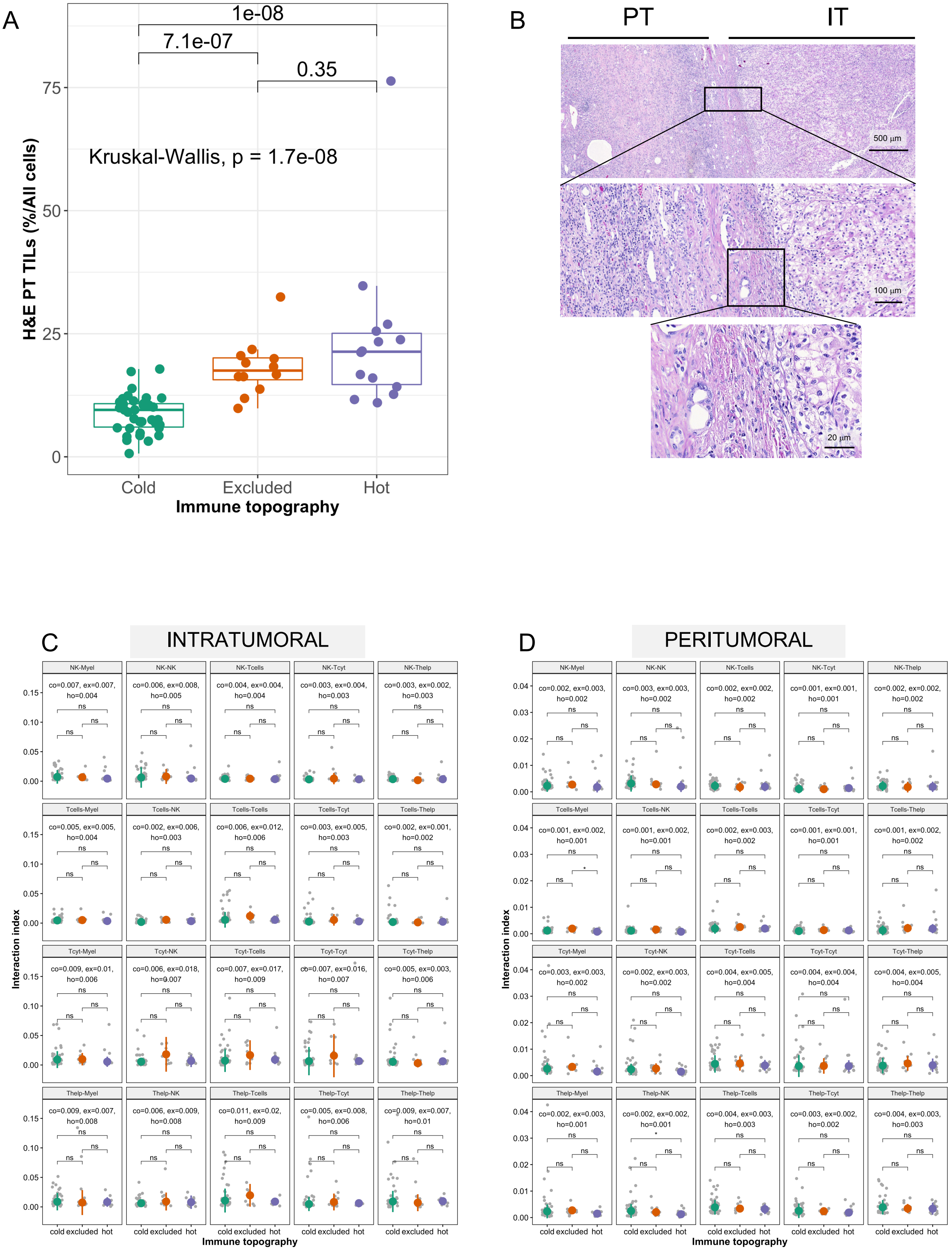

Supplementary Figure 9. (A) Scatter and box plot and Kruskal-Wallis comparison of the peritumoral (PT) tumor-infiltrating lymphocyte (TIL) proportion by immune topographies. (B) Representative H&E-stained image of renal cell carcinoma visualizing the fibrotic stroma at the tumor invasive margin. (C) Panel of cellular interaction frequencies by immune topographies in the IT and (D) PT region. Comparisons have been calculated with Wilcoxon test and p-values adjusted with Benjamini&Hochberg correction. Interaction index represents the proportion of cell1 interacting with cell2 normalized by the total proportion of cell1 and cell2.

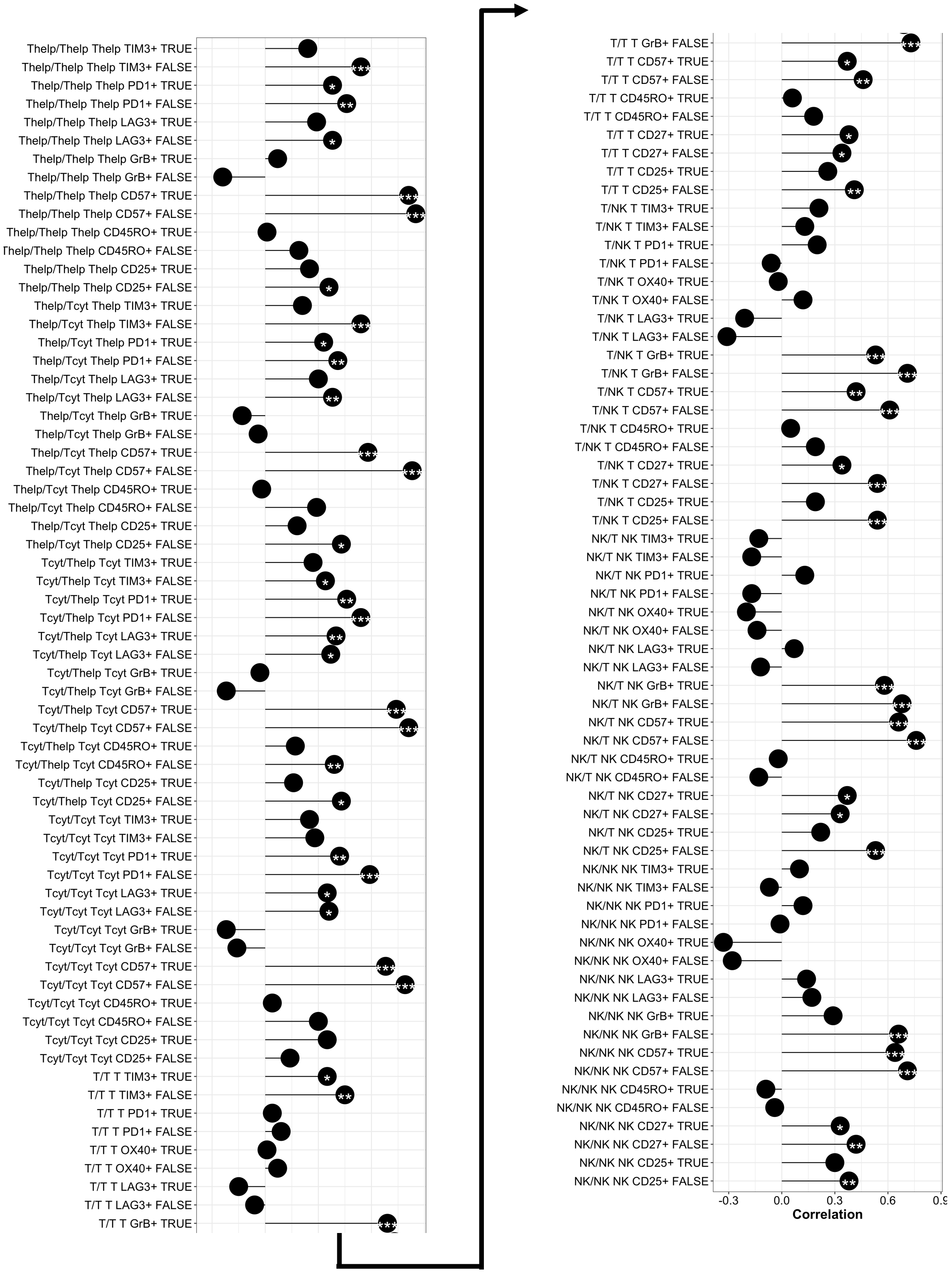

Supplementary Figure 10. Spearman correlation of immunophenotypes by their interacting immune cell pair between IT and PT regions. For instance, Thelp/Tcyt Thelp GrB+ TRUE corresponds to the correlation between IT and PT regions for the expression of granzyme B in helper T cells interacting with cytotoxic T cells. Thelp/Tcyt Thelp GrB+ FALSE corresponds to the same phenotype in non-interacting helper T cells.

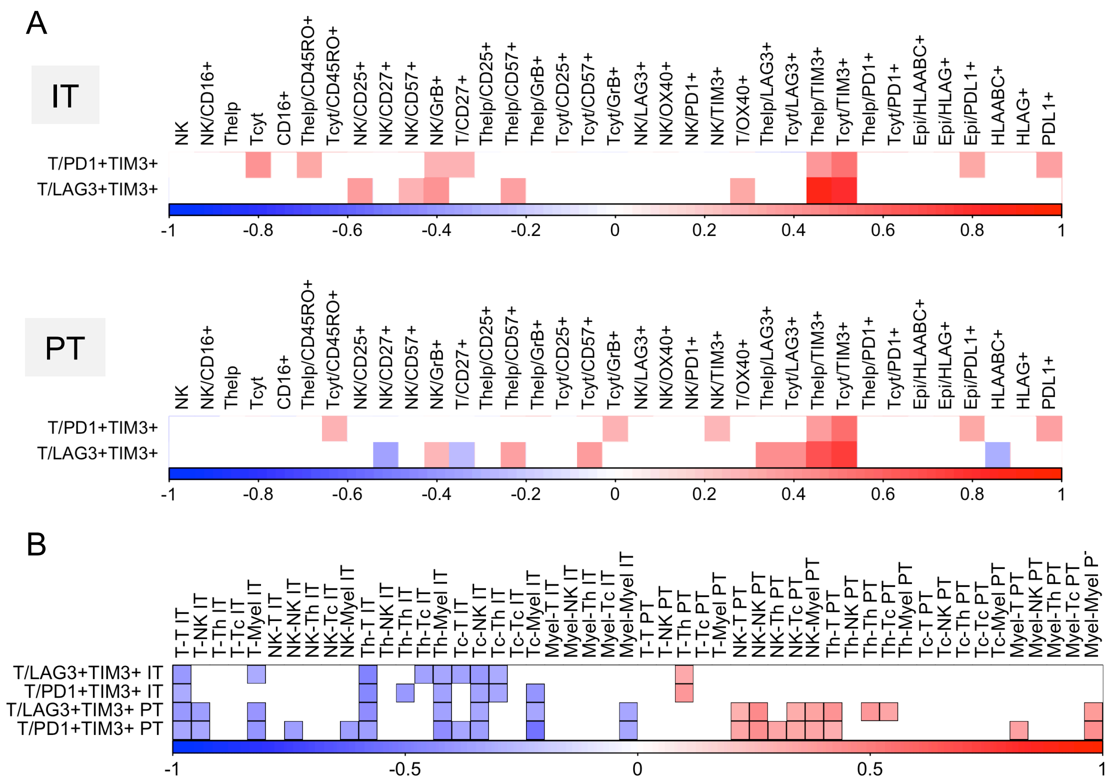

Supplementary Figure 11. (A) Spearman correlation matrix of intratumoral (IT, upper) and peritumoral (PT, lower) immunophenotypes. The color scaling represents the correlation coefficient. P-values have been adjusted with Benjamin & Hochberg correction. Only significant correlations (adjusted p-value <0.05) are color-labelled. Significance: *** p<0.001, ** p<0.01, * p<0.05. (B) Spearman correlation matrix of IT and PT T cell immune checkpoint expression (rows) and cell pair interaction proportion (columns).
